# *In situ* phase transitional polymeric vaccines for improved immunotherapy

**DOI:** 10.1093/nsr/nwab159

**Published:** 2021-08-27

**Authors:** Jie Wang, Yi Wang, Shenglin Qiao, Muhetaerjiang Mamuti, Hongwei An, Hao Wang

**Affiliations:** CAS Center for Excellence in Nanoscience, CAS Key Laboratory for Biomedical Effects of Nanomaterials and Nanosafety, National Center for Nanoscience and Technology (NCNST), Beijing 100190, China; Center of Materials Science and Optoelectronics Engineering, University of Chinese Academy of Sciences, Beijing 100190, China; CAS Center for Excellence in Nanoscience, CAS Key Laboratory for Biomedical Effects of Nanomaterials and Nanosafety, National Center for Nanoscience and Technology (NCNST), Beijing 100190, China; Center of Materials Science and Optoelectronics Engineering, University of Chinese Academy of Sciences, Beijing 100190, China; CAS Center for Excellence in Nanoscience, CAS Key Laboratory for Biomedical Effects of Nanomaterials and Nanosafety, National Center for Nanoscience and Technology (NCNST), Beijing 100190, China; Center of Materials Science and Optoelectronics Engineering, University of Chinese Academy of Sciences, Beijing 100190, China; CAS Center for Excellence in Nanoscience, CAS Key Laboratory for Biomedical Effects of Nanomaterials and Nanosafety, National Center for Nanoscience and Technology (NCNST), Beijing 100190, China; Center of Materials Science and Optoelectronics Engineering, University of Chinese Academy of Sciences, Beijing 100190, China; CAS Center for Excellence in Nanoscience, CAS Key Laboratory for Biomedical Effects of Nanomaterials and Nanosafety, National Center for Nanoscience and Technology (NCNST), Beijing 100190, China; Center of Materials Science and Optoelectronics Engineering, University of Chinese Academy of Sciences, Beijing 100190, China; CAS Center for Excellence in Nanoscience, CAS Key Laboratory for Biomedical Effects of Nanomaterials and Nanosafety, National Center for Nanoscience and Technology (NCNST), Beijing 100190, China; Center of Materials Science and Optoelectronics Engineering, University of Chinese Academy of Sciences, Beijing 100190, China

**Keywords:** nanomedicine, lymph node delivery, cancer vaccine, size modulation, dendritic cell

## Abstract

Cancer vaccines have exhibited immense potential in cancer treatment. Through activating the host's immune system, vaccines stimulate extensive functional T cells to eliminate cancer. However, the therapeutic efficacy of cancer vaccines is limited by their inferior lymph node delivery and inadequate uptake of dendritic cells. Herein, we propose an *in situ* phase transitional strategy on vaccine manufacturing to maximally enhance lymph node drainage while ensuring adequate dendritic cell uptake. The phase transitional vaccines, with dynamic size modulation property, retain a small size (24.4 ± 3.1 nm) during lymph node draining then transform into larger particles (483.0 ± 41.6 nm) on-site by external signal input. Results show that this strategy induced rapid and robust immune response in a mouse melanoma tumor model. Furthermore, a stronger humoral immune response was observed in mice when immunized with MHC-II restricted antigen, which demonstrated that lymph node-targeted cancer vaccine delivery could be effectively manipulated through dynamic size modulation.

## Teaser

By analyzing the unique features of the vaccine delivery process, a new strategy is proposed to improve both lymph node drainage and dendritic cell endocytosis through dynamic size modulation.

## INTRODUCTION

Vaccines aim to initiate host immunity and activate antigen-specific adaptive immune responses to elicit antibodies and expand T cells [1,2]. In recent years, robust antitumor responses mediated by cancer vaccines have shown enormous potential for cancer prevention and regression [3–7]. Cancer vaccines that effectively mobilize immunity against tumor antigens generally originate from lymphocyte priming in lymphoid organs, especially in lymph nodes [8,9]. Lymph nodes, as the primary organ for both innate and adaptive immunity activation, have been extensively exploited as targets for cancer vaccine delivery and potential immune effects have been achieved [10–15]. More importantly, naive T cells must be activated by professional antigen presenting cells (APCs) in lymphoid organs [16,17]. Effectively delivering cancer vaccines to the lymph nodes reduces antigen tolerance and improves safety and biocompatibility [18–21]. Strategies aiming to improve lymph node delivery of cancer vaccines can be attributed to two major pathways: (1) active targeting through antibodies or recruitment molecules [9,10,22,23], and (2) passive targeting using characteristics of delivery systems, such as shape and surface charge [24–26]. Despite such advances, cancer vaccine delivery remains challenging in terms of limited lymphatic draining and inadequate antigen uptake by lymph node-resident dendritic cells, failing to produce a valid antitumor response and weakening the antitumor efficacy.

Cancer vaccines targeting lymph nodes are critical to initiate specific antitumor immune responses. However, their efficacy is substantially limited by their hydrodynamic size, resulting from the unique and complex lymphoid structure [27–30]. A size between 10 and 100 nm would be favorable for lymphoid draining, with larger sizes tending to become trapped within interstitial matrix and smaller sizes penetrating into blood circulation [31,32]. Besides the lymphatic draining, size also has an impact on the endocytosis behavior of dendritic cells in subsequent steps. Dendritic cells can efficiently take up substances between 50 and 500 nm in a size-dependent manner [26,33–35]. In this range, the larger hydrodynamic size promotes higher internalization and dendritic cell activation, which is not favorable for lymphatic draining. As a result, cancer vaccines with unique sizes that meet the requirements for both enhancing lymphatic draining and dendritic cell uptake are desirable prospects for development.

**Figure 1.**
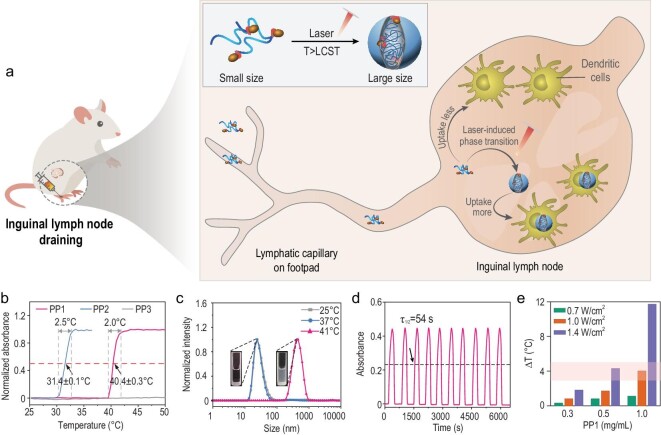
*In situ* phase transitional cancer vaccines. (a) Illustration of *in situ* phase transitional cancer vaccines that enter lymphatic vessels with small hydrodynamic size and transform into larger particles at the lymph node after laser irradiation to improve cancer vaccine efficacy. (b) UV-Vis absorbance of PP1, PP2 and PP3 at 25–50°C. (c) Size distribution of PP1 at 25°C, 37°C and 41°C by DLS. Inset, representative images of the turbidity changes. (d) The transition process of PP1 detected by UV-Vis. τ_1/2_ was defined as the time at which a half-maximal absorbance was detected. (e) Laser-induced temperature increase of PP1 in PBS under different concentrations and laser intensities. The inserted box means appropriate range for temperature increase.

In the current study, we propose an *in situ* phase transitional strategy employing an antigen-linked thermoresponsive polymer to simultaneously enhance lymphatic drainage and lymph node-resident dendritic cells uptake, and stimulate more effective activation of T cells. Previously, it was reported that thermoresponsive poly(N-isopropylacrylamide) (PNIPAM) exhibited a significant size change after stimulation and the transition process is sensitive and controllable, indicating that PNIPAM is an ideal stimuli-responsive carrier material [36–39]. Therefore, we utilized the unique features of PNIPAM and fabricated a phase transitional cancer vaccine with dynamic size modulation property. The phase transitional cancer vaccine is composed of three parts: a thermoresponsive polymer backbone to control the size responsive to temperature changes; a photothermal conversion molecule (cyanine) to convert light into heat and also for *in vivo* tracing; and an antigen peptide (OVA_257–264_) covalently linked to the polymer backbone to estimate specific antitumor immunity (Fig. 1a). We chose a safe phase transition temperature between 37°C and 41°C to ensure the cancer vaccines retained a smaller size during lymph node draining and to reduce the availability of non-specific uptake. Although the phase transition temperature is slightly higher than normothermia, it will not significantly impact the body [40]. Once arrival at the lymph nodes, the vaccine undergoes a sharp collapse with an enlarged size formation under laser-induced photothermal conversion of cyanine. The increased hydrodynamic size assures efficient endocytosis by lymph node-resident dendritic cells, ultimately achieving rapid and sufficient antitumor effects. As vaccine adjuvants are broadly defined by their ability to enhance the specific immune response of antigens [41], manipulating cancer vaccines with our strategy resulted in improved antitumor efficacy as a result of the reinforced lymph node draining and higher engulfment of antigens by dendritic cells, giving this strategy promising adjuvant effects. Collectively, we demonstrated that employing this *in situ* phase transitional strategy in vaccine manipulation could activate dendritic cells and enhance specific T cell activation by enhancing both lymph node draining and dendritic cells engulfment, finally contributing to tumor control and promising therapeutic efficacy.

## RESULTS AND DISCUSSION

### Synthesis and characterization of phase transitional cancer vaccines

We synthesized the thermoresponsive vaccine backbone by classical reversible addition-fragmentation chain transfer (RAFT) polymerization, in which antigen peptide OVA_257–264_ (SIINFEKL) and photothermal conversion molecule cyanine dye were covalently conjugated to the thermoresponsive polymer backbone (Figs S1–S4). By regulating the molar ratio of hydrophobic monomer *N*-isopropylacrylamide (NIPAM) and hydrophilic *N*-(2-hydroxyethyl)acrylamide monomer (HEAM) in PP1 (NIPAM : HEAM = 5 : 1), we successfully obtained a thermoresponsive polymer with an ideal low critical solution temperature (LCST) of 40 ± 0.3°C (Fig. 1b). This polymer maintains coil status at physiological temperature, which is lower than its LCST. As shown in Fig. 1b, PP1 also exhibited a narrow responsive temperature range (2.0–2.5°C). This sharp responsive temperature ensured the biological application possibility for the precisely controlled size changes during vaccine delivery. We also synthesized control groups PP2 and PP3 (Figs 1b and S5–S8) with different LCST. The LCST of PP2 is 31 ± 0.1°C, which maintains a condensate structure with large hydrodynamic size at physiological conditions, and PP3, which is unchanged in the range 25–50°C, implying that PP3 exhibits coil status with small hydrodynamic size between 37–41°C. The structural difference among PP1, PP2 and PP3 was their thermoresponsive backbone. We chose PP3 and PP2 to serve as controls because (i) their hydrodynamic sizes correspond to the initial (small size) and final states (large size) of PP1, respectively; and (ii) to exclude other factors that may influence lymph node draining, such as surface charge. Next, we used dynamic light scattering (DLS) to study and validate the hydrodynamic sizes of PP1, PP2 and PP3 (Fig. 1c). The LCST of PP1 is higher than physiological temperature, therefore, the hydrodynamic size of PP1 was maintained at 24.4 ± 3.1 nm at temperatures <37°C and increased to 483.0 ± 41.6 nm at 41°C. Obviously, The hydrodynamic size at T < LCST stayed within the range 10–30 nm regardless of concentration. Combined with negative results in the critical micelle concentration (CMC) experiments and morphology by TEM, it was indicated that PP1 exhibits multiple coil status without formed micelles structure when T < LCST (Figs S9 and S11a). The PP1 solution in turbidity changed from clear to murky on heating (Fig. 1c, inset), indicating that PP1 retained its coil status at 37°C, then transformed into large particles after phase transition at temperatures > LCST. Considering that concentrations may influence size change behavior, we further analyzed the particle size of PP1 at different concentrations (Fig. S10). PP1 can phase transit at a concentration as low as 0.005 mg/mL (Fig. S9a). The aggregate size is unchanged when the concentration is <1.0 mg/mL. Also, we observed the morphology of PP1 through TEM and scanning electron microscopy (SEM). PP1 presented undefined coiled morphology at room temperature and collapsed in spherical-like particle morphology at temperatures higher than its LCST (Fig. S11). Next, we further investigated the phase transitional sensitivity and stability features of PP1. It was shown that it could collapse within 1 minute, and that it exhibited good recyclability (Fig. 1d). To ensure the phase transition of PP1 by cyanine-induced temperature modulation, we next investigated the photothermal conversion effect of cyanine in PP1 in phosphate buffer saline (PBS) solution. We monitored the real-time temperature changes using forward looking infra-red (FLIR) with 808 nm near-infrared laser irradiation (Figs 1e and S12). PP1 successfully converted light into heat, and the solution temperature was gradually increased along with upregulating the PP1 concentration and radiation intensity (Fig. 1e). To ensure the phase transition process could happen and is safe for the body, we chose a concentration of 0.5 mg/mL at 1.4 W/cm^2^ in the subsequent experiments. Under these conditions, PP1-induced cell cytotoxicity and light-induced toxicity were negligible (Fig. S13).

### Phase transitional cancer vaccines increase uptake of dendritic cells

**Figure 2.**
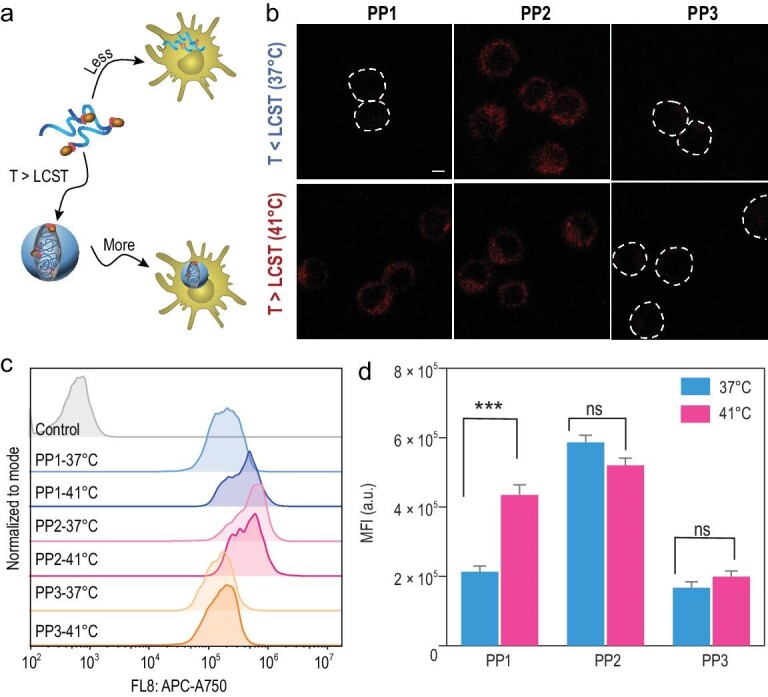
Phase transitional cancer vaccines increase dendritic cell uptake. (a) Schematic illustration of dendritic cell uptake process. (b) Representative uptake images of DC2.4 after incubation with PPs for 30 minutes at 37°C or 41°C. Scale bar: 5 μm. Data analysis of PPs uptake in DC2.4 by (c) flow cytometry and (d) quantification (n = 3). Data were performed as mean ± S.D. (n = 3) and were analyzed by student's t-test. ^***^P < 0.001; ns, not significant.

Efficient endocytosis by antigen presenting cells is crucial for adequate adaptive immune response activation. Thus, we next investigated endocytosis efficacy at various hydrodynamic sizes (Fig. 2a). Firstly, we incubated DC2.4 cells with vaccines at different temperatures for 30 minutes to ensure the size increase of PP1 induced by phase transition. Next, dendritic cell uptake was investigated visually by confocal laser scanning microscopy (CLSM) and quantified with flow cytometry (FACS). Enhanced uptake of PP1 was directly observed after phase transition, whereas uptake of vaccines with small hydrodynamic size was inadequate (Fig. 2b). However, as we controlled particle size through temperature modulation, to exclude the possibility that increasing temperature may impact the endocytosis efficacy, we simultaneously tested the control groups, PP2 and PP3, at 37°C and 41°C (Fig. 2b). The results confirmed that the differences in endocytosis are largely caused by the size increase rather than temperature-induced endocytosis. Moreover, the FACS results further quantitatively confirmed that the phase transition-induced size increase directly improved endocytosis of PP1 (Fig. 2c and d). Exploration of the endocytosis mechanisms found that the uptake was dependent on energy and clathrin (Fig. S14). We concluded that employing the phase transitional strategy in vaccines by increasing the hydrodynamic size could lead to higher endocytosis in dendritic cells, which may benefit for dendritic cells activation and subsequent immune response.

### Phase transitional cancer vaccines initiate specific antitumor immunity

**Figure 3.**
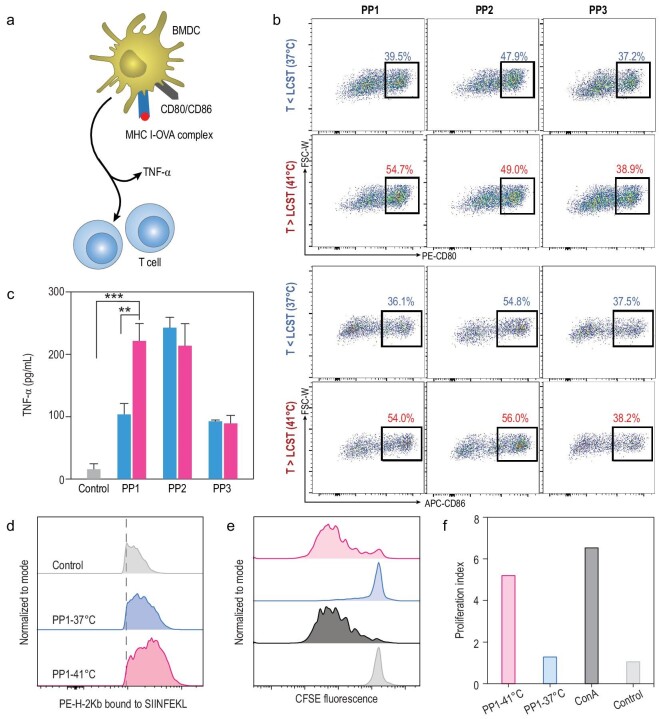
Specific antitumor immunity initiation. (a) Schematic illustration of BMDC maturation and T cell proliferation. (b) CD80 and CD86 expression on BMDCs after treatment with PPs. T > CLST were investigated at 41°C while T < LCST were analyzed at 37°C. (c) TNF-α secretion from BMDC supernatants after incubation with PPs. (d) Flow cytometry analysis of antigen presentation in BMDCs. (e) Representative histogram of T cell proliferation and (f) proliferation index after co-incubating T cells with BMDCs treated with PPs. ConA, concanavalin A at 2 μg/mL.

To identify that enhanced immune response activation resulted from efficient uptake by antigen-presenting cells, bone marrow-derived dendritic cell (BMDC) activation and maturation were measured by treatment with PPs (Fig. 3a), including changes in expression of co-stimulatory factors (CD80 and CD86) on dendritic cells and cytokine secretion, as well as presentation of the antigen-specific MHC I complex (H-2K^b^-SIINFEKL). Firstly, we observed that both CD80 and CD86 were highly expressed under PP1 treatment at temperatures >LCST (Figs 3b and S15), which implied that phase transitional PP1 conversion to larger size contributed to higher uptake and stronger activation of dendritic cells. Similar to PP1 after phase transition, PP2, which stays collapsed at large size, also enabled activation of dendritic cells. However, PP3 did not stimulate dendritic cells effectively because of its smaller size and insufficient uptake (Fig. 3b). It is believed that the unique phase transitional property of PP1, which leads to higher dendritic cell uptake, directly contributes to higher expression of co-stimulatory molecules, instead of temperature changes. The pro-inflammatory cytokine TNF-α, which is crucial for enhancing cellular immune response, was also examined by enzyme-linked immunosorbent assay (ELISA). The phase transitional vaccine promoted robust secretion of TNF-α by BMDCs, indicating the capability of activating higher immune response (Fig. 3c). We further measured the expression of antigen-specific MHC I molecules by FACS to analyze antigen presentation. It was confirmed that the phase transitional cancer vaccines induced prominent specific antigen presentation (Figs 3d and S16). The mean fluorescence intensity exhibited significant enhancement in H-2K^b^-SIINFEKL presentation (Fig. S17). This indicated that the intrinsic antigen process by proteasome or enzymes and cross presentation subsequently led to successful H-2K^b^-SIINFEKL presentation. Significant differences in antigen presentation may be caused by the stark contrast of endocytosis. Above all, these results displayed that the phase transitional vaccine was capable of enhancing the uptake behavior of dendritic cells, which subsequently induced their maturation and higher antigen presentation.

To identify that phase transitional vaccines are able to expand specific antitumor T cells, we explored cytotoxic T cell proliferation stimulated by mature dendritic cells. T cells from an OT-I mouse were previously labeled with carboxyfluorescein succinimidyl amino ester (CFSE) and co-incubated with BMDCs pre-stimulated with phase transitional vaccine for 5 days to evaluate the T cell proliferation [42]. As shown in Fig. 3e, BMDCs pre-treated with phase transitional cancer vaccine can induce significant T cell proliferation. Quantitatively analyzing the proliferation index, this was about five times higher than for the control group (Figs 3f and S18). Taken together, the phase transition-induced size increase can enhance cancer vaccine uptake by dendritic cells, which is beneficial to induce higher BMDC maturation, antigen presentation and subsequent T cell proliferation.

### Phase transitional cancer vaccines promote rapid lymph node drainage and dendritic cell maturation *in vivo*

**Figure 4.**
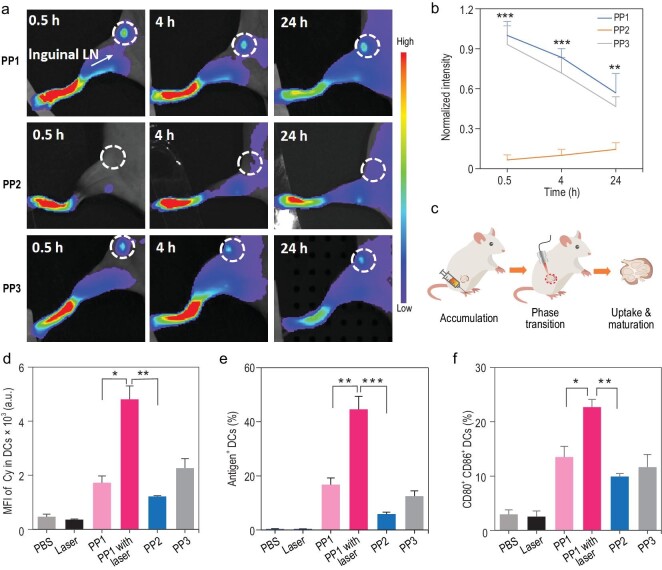
Enhanced lymph node drainage and dendritic cell maturation *in vivo*. (a) Representative images illustrating distribution at various time points. (b) Relative quantification analysis of PPs in draining lymph nodes. (c) Schematic illustration of *in situ* phase transition enhancing lymph node drainage and lymph node-resident dendritic cell uptake *in vivo*. (d) Mean fluorescence intensity of PPs uptake by dendritic cells in lymph node after laser irradiation. (e) Antigen^+^ dendritic cells in lymph node within 2 hours. (f) CD80 and CD86 expression percentages of dendritic cells in lymph node. Data were performed as mean ± S.D. (n = 3) and were analyzed by student's t-test. ^*^P < 0.05; ^**^P < 0.01; ^***^P < 0.001.

Based on the positive effects of phase transitional vaccines on adaptive immunity stimulation *in vitro*, we attempted to identify whether the phase transitional vaccines can improve lymph node drainage and dendritic cell uptake *in vivo*. We first subcutaneously injected cancer vaccines into mice footpad and observed lymph node drainage by fluorescence imaging at various time points. As seen in Fig. 4a and b, the cancer vaccines with small hydrodynamic size (PP1 and PP3) exhibited rapid and efficient lymph node drainage at 0.5 hours and accumulated in lymph nodes until 24 hours compared with the PP2 group, which were mostly trapped at the footpad because of their relatively larger size (483.0 ± 41.6 nm). Although PP2 exhibited limited lymph node drainage, their accumulation in lymph nodes gradually increased, possibly caused by the peripheral uptake of dendritic cells and then migrated into lymph nodes. Except for rapid and higher lymph node drainage, which is related to appropriately small size, long-term accumulation was associated with higher endocytosis potential by lymph node-resident dendritic cells. Therefore, we further evaluated the lymph node-resident dendritic cell uptake and maturation *in vivo* with our phase transitional vaccines. To verify whether the phase transitional cancer vaccines could transform into large particles at lymph nodes *in vivo*, we input near-infrared laser irradiation at inguinal lymph nodes after 30 minutes of phase transitional cancer vaccine injection (Fig. S19) and confirmed the temperature would increase rapidly around the LCST of PP1, which ensured that PP1 could collapse into larger size at lymph nodes *in vivo* after laser irradiation. The minimal aggregated concentration *in vivo* was quantified at ∼0.79 mg/mL (Fig. S20), which is much higher than in *in vitro* studies (Fig. S10). Subsequently, we dissected the inguinal lymph nodes to assess cancer vaccine endocytosis at 2 hours and analyze dendritic cell maturation *in vivo* after 24 hours (Fig. 4c). The fluorescence intensity of the transitional group (PP1 with laser) that could form large particles was significantly enhanced compared with other groups (Fig. 4d). Analyzing the phase transitional vaccine in lymph node-resident dendritic cells, almost 40% of vaccines were taken up within 2 hours (Fig. 4e). Compared with cell-mediated antigen trafficking and other directed lymph node-targeting vaccines, the phase transitional cancer vaccines have more rapid and higher level uptake in lymph nodes [43]. Co-stimulatory factors CD80 and CD86 in dendritic cells showed limited expression at smaller size without phase transitional ability groups (PP1 without laser and PP3) and in the PP2 group, which, in large part, was trapped at the injected site because of poor lymph node draining (Fig. 4f). On the contrary, the phase transitional group of PP1 with laser irradiation showed increased CD80 and CD86 expression and improved the quantity of CD11c^+^ dendritic cells in lymph nodes (Figs 4f and S21). Taken together, the results indicate that increasing lymph node drainage and promoting lymph node-resident dendritic cell activation can be achieved by cancer vaccines with our phase transitional strategy, achieving a more effective and rapid adaptive immune response. Moreover, enhanced humoral immunity against antigen peptide OVA_323–339_ was conducted, which further proved the efficacy and applicability of our strategy (Fig. S22).

### Phase transitional cancer vaccine increases CD8^+^ T cell response and inhibits tumor growth

**Figure 5.**
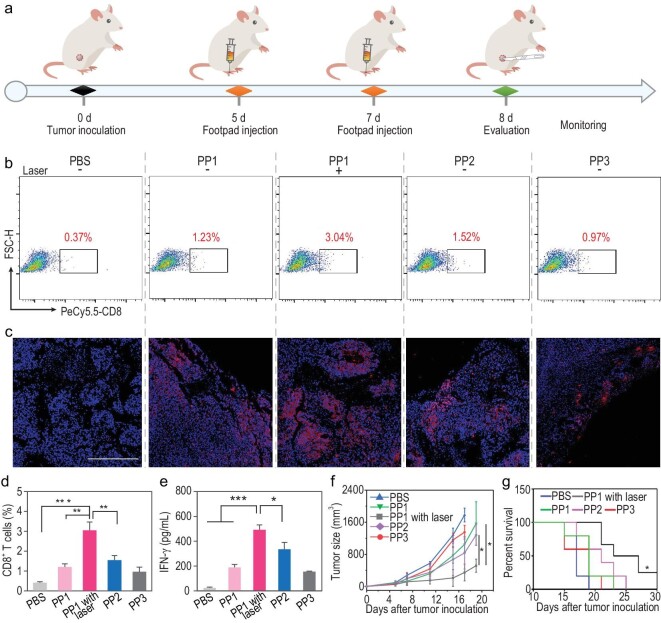
Enhanced anti-tumor immune response of phase transitional cancer vaccines (a) Schematic illustration of antitumor therapy. (b) Representative flow dot plots of CD8^+^ T cells in tumor tissue and quantified by flow cytometry in (d). (c) Representative immunofluorescence images of tumor tissues under various treatments. Scale bar: 100 μm; blue, nucleus; red, CD8^+^ T cells. (e) IFN-γ secretion in tumor tissue with indicated treatment by ELISA. (f) Tumor growth inhibition and (g) survival under various treatments of B16-F10-OVA tumor model in C57BL/6 mice (n = 5). Data were analyzed by student's t-test. ^*^P < 0.05, ^**^P < 0.01, ^***^P < 0.001. The survival curve analysis was compared using Kaplan–Meier curves followed by the log-rank test. ^*^P < 0.05.

Finally, we evaluated the therapeutic efficacy of the cancer vaccine with our phase transition strategy (Fig. 5a). C57BL/6 mice were subcutaneously inoculated with B16-F10-OVA tumor cells on their right flank. On day 5, the mice were immunized subcutaneously at the footpad with two doses (1 day apart) of vaccine followed by laser irradiation at the inguinal lymph node site three times for 30 minutes. On day 8, we analyzed the frequency of CD8^+^ T cells in tumor tissue (Fig. 5b and c; Fig. S23). The PP1 without a laser irradiation group and the PP3 group showed minimal induction of CD8^+^ T cell infiltration in tumor tissue because of their non-phase transition capability. This blunted immune response activation, while the PP2 group exhibited only slightly higher CD8^+^ T cell infiltration, which resulted from uptake by peripheral dendritic cells and migration into the lymph nodes. However, this was still lower than the phase transitional group (PP1 with laser irradiation), which generated strong CD8^+^ T cell infiltration (4.3-fold greater than the PBS group, *P* = 0.0004 and 1.8-fold greater than the PP2 group, *P* = 0.0053, Fig. 5d). To assess the tumor-infiltrated T cell function, we measured IFN-γ secretion in the tumor tissue. PP1 with the laser group led to significantly higher IFN-γ secretion compared with the control groups (*P*< 0.0001 compared with the PBS group, *P* = 0.0003 compared with the PP1 group, *P* = 0.0336 compared with the PP2 group) (Fig. 5e). Finally, we monitored tumor growth to evaluate the therapeutic efficacy (Fig. 5f and g). The phase transitional cancer vaccine group (PP1 with laser) retarded the tumor growth compared with larger size PP2 (*P* = 0.0263) and with the PP1 group without phase transition (*P* = 0.0279). It also significantly prolonged the survival time of mice (*P* = 0.0271). Moreover, we did not observe any weight loss or major organ toxicity after treatment with vaccines (Fig. S24), which demonstrated the biocompatibility and biosafety of PPs. Taken together, the cancer vaccines based on this *in situ* phase transitional strategy exhibited promising therapeutic efficacy in inhibiting tumor progression. The results demonstrated that cancer vaccines with improved lymph node draining and antigen utilization mediated by this strategy were highly beneficial for inducing an adequate antigen-specific immune response.

## CONCLUSION

Preclinical studies of nano/biomaterials have indicated their potential in tumor-targeting or immune organ-targeting strategies, and achieved promising therapeutic outcomes while reducing toxicities/side effects [44]. Lymph nodes are the most widely investigated target for therapeutic cancer vaccines and other immunotherapeutics. Antigen immunogenicity could be enhanced significantly by increasing antigen utilization and decreasing off-target immune responses. The unique structure of the lymphatic system and the size-restricted nature of lymph node drainage has directed design of various nano/biomaterials. In this work, we fully utilized the thermoresponsive and size change of PNIPAM polymer, and developed *in situ* phase-transitional strategy manufacturing cancer vaccines with improved lymph node draining efficacy and higher dendritic cell uptake, which represent two prerequisites for adaptive immune response initiation. Our previous work has proved this phase transitional vaccine backbone has excellent responsive performance and satisfactory biocompatibility [38]. Moreover, *in situ* manipulation of dendritic cells has also achieved promising therapeutic efficacy in solid cancer [45,46]. In this work, we investigated the possibility of vaccine size dynamic control in guidance of the phase transitional strategy in the tumor model by modulating the physiochemical properties of vaccine composition, which generated promising therapeutic efficacy in suppressing tumor growth. Basically, adjuvants themselves exert an immunostimulation effect through pathogen-associated molecular patterns (PAMPs) receptors such as Toll-like receptors (TLRs). The phase transitional nanoparticles as exogenous components can be recognized by the host immune system. Meanwhile, the phase transitional feature favors desired endocytosis and accumulation in host immune cells, which amplify the specific response of antigens. Moreover, it also fits for antigen proteins or antigen proteins with adjuvant delivery through interaction with a functional group, or combined with other immunotherapies such as checkpoint blockade, which may indicate high potential for further immunotherapy combinations and clinical translation.

## METHODS AND MATERIALS

The experimental details are given in the Supplementary data.

## Supplementary Material

nwab159_Supplemental_File

## References

[1] Rappuoli R , Mandl CW, Black S et al. Vaccines for the twenty-first century society. Nat Rev Immunol 2011; 11: 865–72. 10.1038/nri3085 22051890 PMC7098427

[2] Finn OJ. The dawn of vaccines for cancer prevention. Nat Rev Immunol 2018; 18: 183–94. 10.1038/nri.2017.140 29279613

[3] Zhang LX , Sun XM, Jia YB et al. Nanovaccine's rapid induction of anti-tumor immunity significantly improves malignant cancer immunotherapy. Nano Today 2020; 35: 100923. 10.1016/j.nantod.2020.100923

[4] Ci T , Li H, Chen G et al. Cryo-shocked cancer cells for targeted drug delivery and vaccination. Sci Adv 2020; 6: eabc3013. 10.1126/sciadv.abc3013 33298439 PMC7725453

[5] Gu Y , Liu YF, Cao XT. Evolving strategies for tumor immunotherapy: enhancing the enhancer and suppressing the suppressor. Natl Sci Rev 2017; 4: 161–3. 10.1093/nsr/nwx032

[6] Wang Y , Zhang L, Xu Z et al. mRNA vaccine with antigen-specific checkpoint blockade induces an enhanced immune response against established melanoma. Mol Ther 2018; 26: 420–34. 10.1016/j.ymthe.2017.11.009 29249397 PMC5835019

[7] Zhao X , Yang K, Zhao R et al. Inducing enhanced immunogenic cell death with nanocarrier-based drug delivery systems for pancreatic cancer therapy. Biomaterials 2016; 102: 187–97. 10.1016/j.biomaterials.2016.06.032 27343466

[8] Yang WJ , Zhou ZJ, Lau J et al. Functional t cell activation by smart nanosystems for effective cancer immunotherapy. Nano Today 2019; 27: 28–47. 10.1016/j.nantod.2019.05.004

[9] Najibi AJ , Mooney DJ. Cell and tissue engineering in lymph nodes for cancer immunotherapy. Adv Drug Deliv Rev 2020; 161: 42–62. 10.1016/j.addr.2020.07.023 32750376 PMC7736208

[10] Liu H , Moynihan KD, Zheng Y et al. Structure-based programming of lymph-node targeting in molecular vaccines. Nature 2014; 507: 519–22. 10.1038/nature12978 24531764 PMC4069155

[11] Gong N , Zhang Y, Teng X et al. Proton-driven transformable nanovaccine for cancer immunotherapy. Nat Nanotechnol 2020; 15: 1053–64. 10.1038/s41565-020-00782-3 33106640 PMC7719078

[12] Liu S , Jiang Q, Zhao X et al. A DNA nanodevice-based vaccine for cancer immunotherapy. Nat Mater 2021; 20: 421–30. 10.1038/s41563-020-0793-6 32895504

[13] Liang F , Lindgren G, Sandgren KJ et al. Vaccine priming is restricted to draining lymph nodes and controlled by adjuvant-mediated antigen uptake. Sci Transl Med 2017; 9: eaal2094. 10.1126/scitranslmed.aal2094 28592561

[14] Sahdev P , Ochyl LJ, Moon JJ. Biomaterials for nanoparticle vaccine delivery systems. Pharm Res 2014; 31: 2563–82. 10.1007/s11095-014-1419-y 24848341 PMC4198431

[15] Xu J , Lv J, Zhuang Q et al. A general strategy towards personalized nanovaccines based on fluoropolymers for post-surgical cancer immunotherapy. Nat Nanotechnol 2020; 15: 1043–52. 10.1038/s41565-020-00781-4 33139933

[16] Weninger W , Manjunath N, von Andrian UH. Migration and differentiation of CD8^+^ T cells. Immunol Rev 2002; 186: 221–33. 10.1034/j.1600-065X.2002.18618.x 12234374

[17] Villablanca EJ , Russo V, Mora JR. Dendritic cell migration and lymphocyte homing imprinting. Histol Histopath 2008; 23: 897–910. 10.14670/HH-23.897 18437688

[18] Hailemichael Y , Dai Z, Jaffarzad N et al. Persistent antigen at vaccination sites induces tumor-specific CD8^+^ T cell sequestration, dysfunction and deletion. Nat Med 2013; 19: 465–72. 10.1038/nm.3105 23455713 PMC3618499

[19] Zhang Y , Pan J, Li H et al. Albumin based nanomedicine for enhancing tacrolimus safety and lymphatic targeting efficiency. J Biomed Nanotechnol 2019; 15: 1313–24. 10.1166/jbn.2019.2777 31072438

[20] Moynihan KD , Holden RL, Mehta NK et al. Enhancement of peptide vaccine immunogenicity by increasing lymphatic drainage and boosting serum stability. Cancer Immunol Res 2018; 6: 1025–38. 10.1158/2326-6066.CIR-17-0607 29915023 PMC6247902

[21] Wang JH , Wang S, Ye T et al. Choice of nanovaccine delivery mode has profound impacts on the intralymph node spatiotemporal distribution and immunotherapy efficacy. Adv Sci 2020; 7: 2001108. 10.1002/advs.202001108 PMC7539204 33042743

[22] Yoo E , Salyer ACD, Brush MJ et al. Hyaluronic acid conjugates of TLR7/8 agonists for targeted delivery to secondary lymphoid tissue. Bioconjugate Chem 2018; 29: 2741–54. 10.1021/acs.bioconjchem.8b00386 29969553

[23] Zhu G , Lynn GM, Jacobson O et al. Albumin/vaccine nanocomplexes that assemble *in vivo* for combination cancer immunotherapy. Nat Commun 2017; 8: 1954. 10.1038/s41467-017-02191-y 29203865 PMC5715147

[24] Jiang H , Wang Q, Sun X. Lymph node targeting strategies to improve vaccination efficacy. J Control Release 2017; 267: 47–56. 10.1016/j.jconrel.2017.08.009 28818619

[25] Kumar S , Anselmo AC, Banerjee A et al. Shape and size-dependent immune response to antigen-carrying nanoparticles. J Control Release 2015; 220: 141–8. 10.1016/j.jconrel.2015.09.069 26437263

[26] Foged C , Brodin B, Frokjaer S et al. Particle size and surface charge affect particle uptake by human dendritic cells in an *in vitro* model. Int J Pharm 2005; 298: 315–22. 10.1016/j.ijpharm.2005.03.035 15961266

[27] Schudel A , Chapman AP, Yau MK et al. Programmable multistage drug delivery to lymph nodes. Nat Nanotechnol 2020; 15: 491–9. 10.1038/s41565-020-0679-4 32523099 PMC7305972

[28] Zhang YN , Lazarovits J, Poon W et al. Nanoparticle size influences antigen retention and presentation in lymph node follicles for humoral immunity. Nano Lett 2019; 19: 7226–35. 10.1021/acs.nanolett.9b02834 31508968

[29] Swartz MA. The physiology of the lymphatic system. Adv Drug Deliv Rev 2001; 50: 3–20. 10.1016/S0169-409X(01)00150-8 11489331

[30] Zhao Y , Guo Y, Tang L. Engineering cancer vaccines using stimuli-responsive biomaterials. Nano Res 2018; 11: 5355–71. 10.1007/s12274-018-2162-1

[31] Bachmann MF , Jennings GT. Vaccine delivery: a matter of size, geometry, kinetics and molecular patterns. Nat Rev Immunol 2010; 10: 787–96. 10.1038/nri2868 20948547

[32] Reddy ST , van der Vlies AJ, Simeoni E et al. Exploiting lymphatic transport and complement activation in nanoparticle vaccines. Nat Biotechnol 2007; 25: 1159–64. 10.1038/nbt1332 17873867

[33] Kim H , Uto T, Akagi T et al. Amphiphilic poly(amino acid) nanoparticles induce size-dependent dendritic cell maturation. Adv Funct Mater 2010; 20: 3925–31. 10.1002/adfm.201000021

[34] He C , Hu Y, Yin L et al. Effects of particle size and surface charge on cellular uptake and biodistribution of polymeric nanoparticles. Biomaterials 2010; 31: 3657–66. 10.1016/j.biomaterials.2010.01.065 20138662

[35] Sousa de Almeida M , Susnik E, Drasler B et al. Understanding nanoparticle endocytosis to improve targeting strategies in nanomedicine. Chem Soc Rev 2021; 50: 5397–434. 10.1039/D0CS01127D 33666625 PMC8111542

[36] Bodelon G , Montes GV, Fernandez LC et al. Au@pNIPAM SERRS tags for multiplex immunophenotyping cellular receptors and imaging tumor cells. Small 2015; 11: 4149–57. 10.1002/smll.201500269 25939486

[37] Qiao SL , Ma Y, Wang Y et al. General approach of stimuli-induced aggregation for monitoring tumor therapy. ACS Nano 2017; 11: 7301–11. 10.1021/acsnano.7b03375 28628744

[38] Ma Y , Qiao SL, Wang Y et al. Nanoantagonists with nanophase-segregated surfaces for improved cancer immunotherapy. Biomaterials 2018; 156: 248–57. 10.1016/j.biomaterials.2017.11.048 29216535

[39] Qiao SL , Wang Y, Lin YX et al. Thermo-controlled *in situ* phase transition of polymer-peptides on cell surfaces for high-performance proliferative inhibition. ACS Appl Mater Interfaces 2016; 8: 17016–22. 10.1021/acsami.6b04580 27348260

[40] Jaque D , Martinez ML, del Rosal B et al. Nanoparticles for photothermal therapies. Nanoscale 2014; 6: 9494–530. 10.1039/C4NR00708E 25030381

[41] O’Hagan DT , Valiante NM. Recent advances in the discovery and delivery of vaccine adjuvants. Nat Rev Drug Discov 2003; 2: 727–35. 10.1038/nrd1176 12951579 PMC7097252

[42] Quah BJC , Warren HS, Parish CR. Monitoring lymphocyte proliferation *in vitro* and *in vivo* with the intracellular fluorescent dye carboxyfluorescein diacetate succinimidyl ester. Nat Protoc 2007; 2: 2049–56. 10.1038/nprot.2007.296 17853860

[43] Irvine DJ , Aung A, Silva M. Controlling timing and location in vaccines. Adv Drug Deliv Rev 2020; 158: 91–115. 10.1016/j.addr.2020.06.019 32598970 PMC7318960

[44] Shi Y. Clinical translation of nanomedicine and biomaterials for cancer immunotherapy: progress and perspectives. Adv Therap 2020; 3: 1900215. 10.1002/adtp.201900215

[45] Jaeyun K , David JM. *In vivo* modulation of dendritic cells by engineered materials: towards new cancer vaccines. Nano Today 2011; 6: 466–77. 22125572 10.1016/j.nantod.2011.08.005 PMC3224090

[46] Wang Y , Lin YX, Wang J *et al.* *In situ* manipulation of dendritic cells by an autophagy-regulative nanoactivator enables effective cancer immunotherapy. ACS Nano 2019; 13: 7568–77. 10.1021/acsnano.9b00143 31260255

