# Supplementary material for: In situ phase transitional polymeric vaccines for improved immunotherapy
Source: Natl Sci Rev. 2021 Aug 27;9(2):nwab159. doi: 10.1093/nsr/nwab159 (PMC8824734; doi:10.1093/nsr/nwab159)
Supplement: nwab159_Supplemental_File [file nwab159_supplemental_file.docx]

**Supporting Information**

***In Situ* Phase Transitional Polymeric Vaccines for Improved Immunotherapy**

Jie Wang^1,2,#^, Yi Wang^1,2,#^, Shenglin Qiao^1,2^, Muhetaerjiang Mamuti^1,2^, Hongwei An^1,2^ and Hao Wang^1,2,^*

1. CAS Center for Excellence in Nanoscience, CAS Key Laboratory for Biomedical Effects of Nanomaterials and Nanosafety, National Center for Nanoscience and Technology (NCNST), Beijing 100190, China
2. Center of Materials Science and Optoelectronics Engineering, University of Chinese Academy of Sciences, Beijing 100190, China

#Equally contributed to this work.

*Corresponding author. E-mail: [wanghao@nanoctr.cn](mailto:wanghao@nanoctr.cn)

1. **Experimental Section**

**1.1. Materials.** N-isopropylacrylamide (NIPAM) was purchased from Aldrich Chemical Corporation (Shang Hai, China), recrystallization by n-hexane three times. 2, 2-azobisisobutyronitrile (AIBN) was purchased from Sigma Aldrich Chemical Corporation and recrystallization in ethanol three times. 2-(Dodecylthiocarbonothioylthio)-2-methylpropionic acid N-hydroxysuccinimide ester (CTA), N-2-Hydroxyethyl acrylamide (HEAm) were purchased from Sigma-Aldrich and HEAm was removal stabilizer by neutral aluminum oxide column. 9-Fluorenylmethocycarbonyl (Fmoc)-protected amino acids and Wang resin were purchased from GL Biochem (China). Cell counting kit-8 (CCK8) were purchased from Beyotime Institute of Biotechnology, China. B16-F10-OVA was gifted from National Center of nanoscience and technology, Xingjie Liang group. Cell culture medium and fetal bovine serum were purchased from ThermoFisher company. Trypsin and Penicillin-Streptomycin were purchased from Wisent company. C57BL/6 animals were purchased from Beijing Vital River Laboratory Animal Technology Co., Ltd. Other solvents and agents were used as received.

**1.2. Synthesis of Phase Transitional Cancer Vaccines.** The thermo-responsive polymers were synthesized by Reversible Addition Fragmentation Chain Transfer Polymerization (RAFT). NIPAM (0.015 mol), HEAM (0.003 mol), CTA (0.092 mmol), were dissolved in 3 mL DMF and degassed with nitrogen 30 min. AIBN (0.018 mmol) dissolved in a small amount of DMF was added under nitrogen, then increased to 65^o^C of oil bath temperature and reacted around 10 h. Reaction product was purified by redissolving in 2 mL THF and precipitated with cold ether. The product was vacuum dried after three times purification. The control polymer was polymerized by only one monomer, NIPAM or HEAm. The solid-phase peptide synthesis method was used to prepared the Dde protected antigen peptide (RDCRDSIINFEK(Dde)L). the polymer peptide was synthesized by condensation reaction between amino in terminal peptide and carboxyl group activated by succinimide in phosphate buffered solution (pH 8.0) for 3 days. The product was purified through dialyzed against deionized water (MWCO: 3500 Da) and vacuum freeze-dried. The final product was obtained after remove Dde group in hydrazine hydrate/DMF (v/v = 1/50) followed with dialyzed against deionized water (MWCO: 3500 Da) and vacuum freeze-dried.

**1.3. NMR Spectra.** The chemical structures of polymers backbone and PPs were examined by ^1^H NMR spectra. Dissolved polymers or PPs (5 - 10 mg) in 6 mL DMSO-*d_6_* and detected using Bruker AVANCE III HD 400 (^1^H, 400 MHz), TMS as an internal standard.

**1.4. Phase Transition Temperature Measurements.** The phase transition temperature was characterized in PBS at concentration of 300 μg/mL, with heating performed at a rate of 1^o^C by UV-vis spectrophotometer equipped with a thermoelectric temperature controller.

**1.5. Dynamic Light Scattering (DLS).** The hydrodynamic diameters and particles size distribution of polymer peptide vaccines were detected using dynamic light scattering (DLS) analyzer (Zetasizer Nano ZS) in PBS with various concentration. The hydrodynamic size of PP1 under phase transitional condition was examined in 41^o^C after the temperature was stabilized.

**1.6. critical micelle concentration (CMC)**. 3 ug pyrene was dissolved in 15 ul acetone and removed the solvent in the dark by natural volatilization. Added 20 ul PP1 with specific concentrations, respectively. The emission spectrum of each solution was recorded with the fluorescence spectrophotometer (λex = 330 nm). Intensity ratio of peak I (λem = 373 nm) and peak II (λex = 384 nm) was calculated and plotted to determine the CMC value (I_373_/I_384_).

**1.7. Transmission Electron Microscopy (TEM).** The morphology was observed using transmission electron microscopy (TEM, Tecnia G2 20 S-TWIN) with 200 kv acceleration voltage. The polymer peptide vaccines (0.5 mg/mL in water) was dropped onto a copper mesh with the surrounding temperature of 41^o^C. Removed excess liquid after 10 min, then stained with uranyl acetate solution for 1 min. The copper mesh was washed three times in deionized water for 30 s and finally dried at room temperature.

**1.8. Photo - Thermal Conversion Analysis.** Samples were dissolved in phosphate buffered solution (pH 7.4) with different concentration (0.3, 0.5, 1.0 mg/mL) in the 1.5 mL Axygen tubes. 50 uL samples were irradiated under various 808 nm laser intensity (0.7, 1.0, 1.4 W/cm^2^) for 5 min, a forward-looking infrared (FLIR) camera was used was monitor the real-time temperature change.

**1.9. Cell Cytotoxicity Assay.** DC2.4 cells were seeded in 96 well plates at a density of 5×10^3^ cells/mL. Various concentrations of PP1, PP2, PP3 or different irradiating times with 1.4 W/cm^2^ were treated with DC2.4. The cells were incubated for another 24 h in a humidified atmosphere with 5% CO_2_ at 37^o^C. After 24 h, the cells were removed supernatant and wash three times using phosphate buffered solution (pH 7.4), then 100 μL RPMI 1640 cell culture medium containing 10% CCK8 was added for each well and incubated for another 2 h. The absorption at 450 nm (testing wavelength) and 690 nm (reference wavelength) was tested using the microplate (Enspire). The results were expressed as: cell viability = (A_sample_ - A_blank_)/(A_control_ - A_blank_)×100%. All experiments were performed in triplicate.

**1.10. Confocal Laser Scanning Microscopy.** DC2.4 cells were cultured in RPMI 1640 medium, containing 10% fetal bovine serum (FBS) and 1% Penicillin-Streptomycin at 37^o^C overnight. The 0.5 mg/mL PP1, PP2 and PP3 were add and incubated in 37^o^C or 41^o^C for 30 min, then removed the supernatant and washed three times with PBS. Finally, cells were imaged using Zeiss LSM710 confocal laser scanning microscope under 63 × oil objective lens.

**1.11. Flow Cytometry Analysis.** Cells were seeded in six-well plates at a density of 1×10^5^ cells per well, and treated with various polymer vaccines for 30 min in 37^o^C or 41^o^C, then removed supernatant and replaced with fresh RPMI 1640 cell culture medium (10% FBS and 1% penicillin-streptomycin) for overnight. The cells were collected and resuspended with antibody containing FACS staining buffer (2% FBS in PBS), staining was incubated on ice and avoid light for 30 min. Then samples were washed with 0.2 mL FACS staining buffer, analyzed by flow cytometry (BD Calibur). Flowjo software was used for data analyzing.

**1.12. Enzyme - Linked Immunosorbent Assay.** The assay was based on double antibody sandwich method. Samples and standard substance were added according to the steps of ELISA kit (Mouse IFN-γ ELISA Kit abs520007; Mouse TNF-α ELISA Kit abs520010). Standard curve was fitting according to the concentration and OD value of standard substance. The sample concentrations were calculated based on the standard curve. For sample preparation, cell culture supernatant was collected by 1500 rpm/min for 3 min and diluted with dilution buffer (1% BSA in PBST). For serum IgG analysis assay, firstly, serum samples were collected from retro-orbital plexus in mice and placed at room temperature for 3 h, then centrifuged for 15 min (1000 g) and collected supernatant, diluted the supernatant at the ratio of 1:10^1^, 1:10^2^, 1:10^3^, 1:10^4^, 1:10^5^ in dilution buffer. Peroxidase-AffiniPure Goat Anti-Mouse IgG (H+L) (Jackson, 115-035-003) were diluted and added 100 μL per well. After incubated for 60 min at 37^o^C, wash 5 times by wash buffer, then the TMB solution and stop solution were added. The absorbance was detected at 450 nm using Enspire device.

**1.13. *In vivo* Imaging.** Animals were hair removal and fasting one day in advance. At imaging, animals were firstly anesthesia with isoflurane and footpad injected with PP1, PP2 or PP3 (5 mg/mL). Using IVIS Spectrum for Optical imaging at 0.5 h, 4 h and 24 h respectively. The inguinal lymph node position indicated for quantitative analysis.

**1.14. Antitumor Activity.** All animal experiments were performed complying with the NIH guidelines for the care and use of laboratory animals of NCNST Animal Study Committees’ requirements and according to the protocol approved by the Institutional Animal Care. The xenograft melanoma tumor cells were established by subcutaneously injecting B16-F10-OVA cells (5×10^6^ cells). At fifth day after tumor implantation, the mice were divided into 5 groups (n = 5) and treated with footpad injection of PP1, PP2 or PP3 with or without laser irradiation at inguinal lymph node site (1.4 W/cm^2^ for 30 min) at 30 min, 4 h, and 12 h respectively. At day 7, same treatment as the fifth day was implemented again. Vernier calipers were used to measure tumor size every other day and observe and record the survival of the mice. Tumor volume was analyzed by the formula: V = 0.5* (L*W^2^), where the length (L) and width (W) was represent the longest and shortest axis of tumors, respectively.

**1.15. Immunofluorescence Assay.** The immunofluorescence assay was conducted as followed and referred in previous work (*ACS Nano* 2020; **14:** 7170-80). Specifically, tissues were collected after treatment and fixed with 4% paraformaldehyde for 24 h followed by paraffin section. The slices were deparaffinized with EDTA buffer in a microwave. Then, 10% normal goat serum was added and blocked 30 min at room temperature, washed with PBS for three times, incubated at 4^o^C overnight with primary antibodies (CD8, CD80, CD86, CD11c). Next, it washed with PBS three times and incubated with fluorescently labeled goat anti-rabbit IgG H&L secondary antibody for 1 h at room temperature. The nuclei were stained with DAPI dye another 30 min at room temperature and washed with PBS. Finally, the sample slides were observed by a confocal microscope (Zeiss LSM710).

**1.16. Statistical Analysis.** All the experiments were performed three times unless specifically mentioned. Data were presented was mean ± standard deviation (SD). Comparison between groups were analyzed with one-way analysis of variance (ANOVA) and two - sample student t-test. Animal survival curve were compared using Kaplan-Meier curves followed by the log-rank test. *p<0.05, **p<0.01, ***p<0.001.

**2. Supporting Figures**


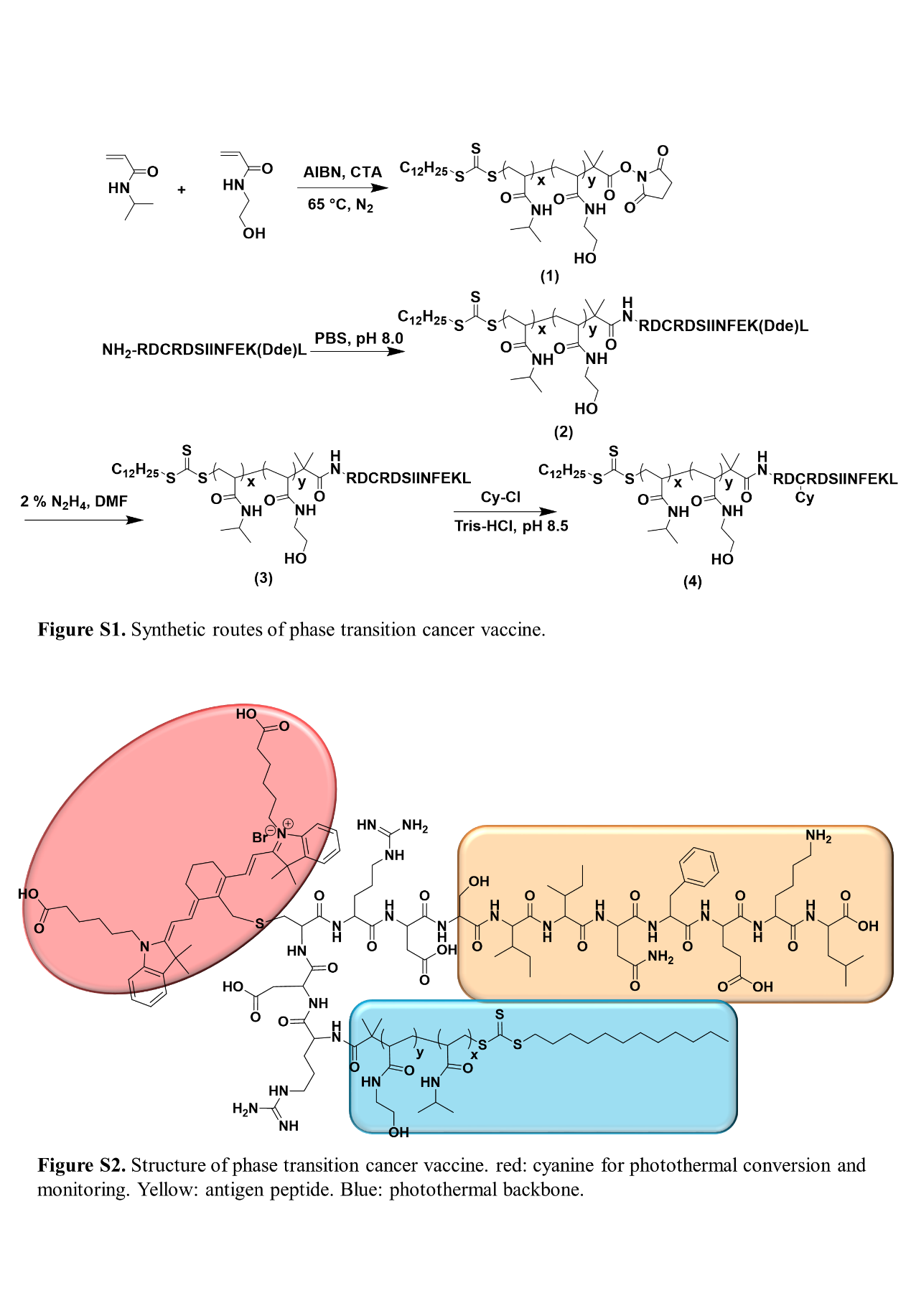


**b**

**a**


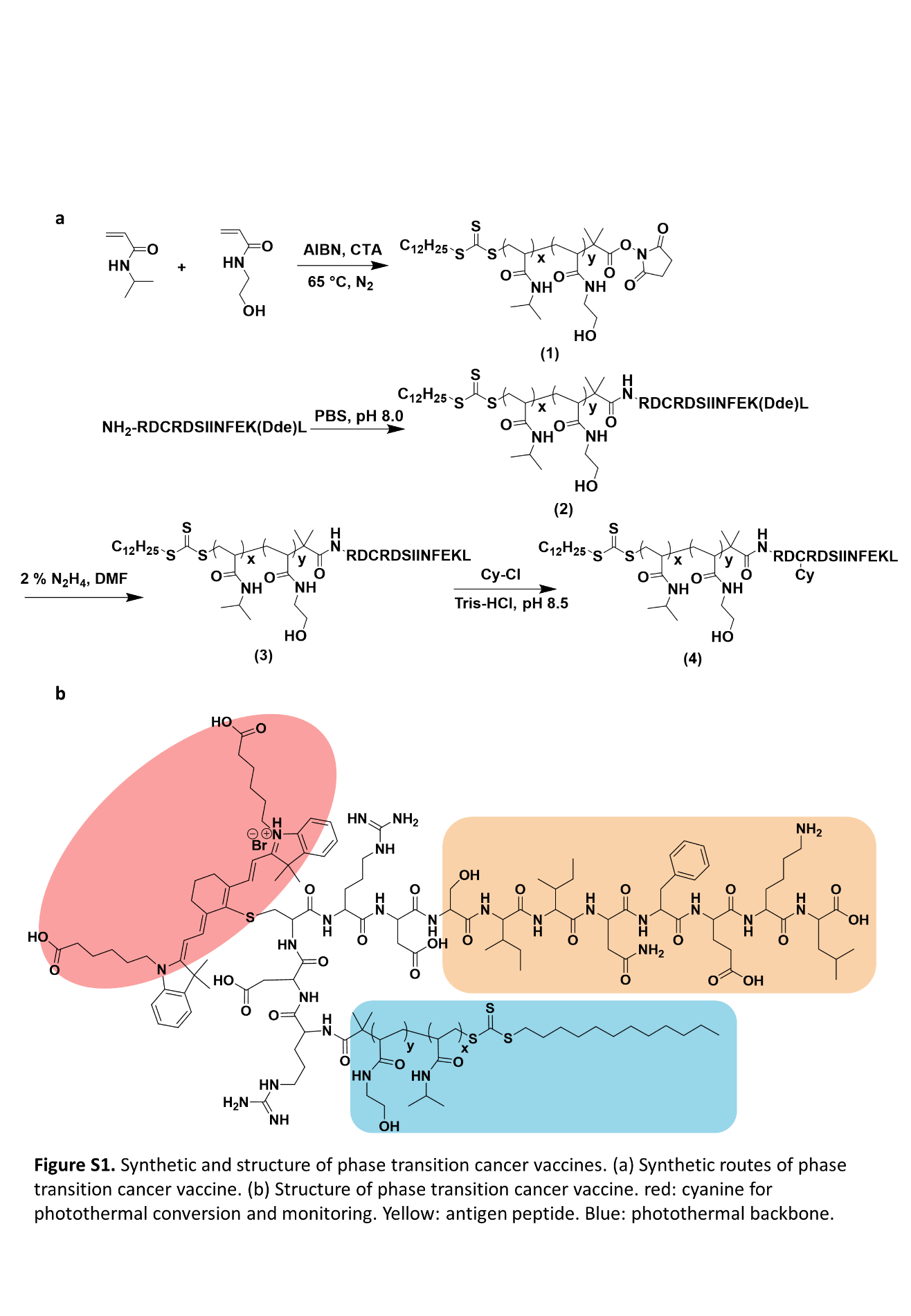


**Figure S1** (a) Synthetic routes of phase transition cancer vaccine. (b) Structure of phase transition cancer vaccine. Red box: cyanine for photothermal conversion and monitoring. Yellow box: antigen peptide. Blue box: thermal-sensitive backbone.


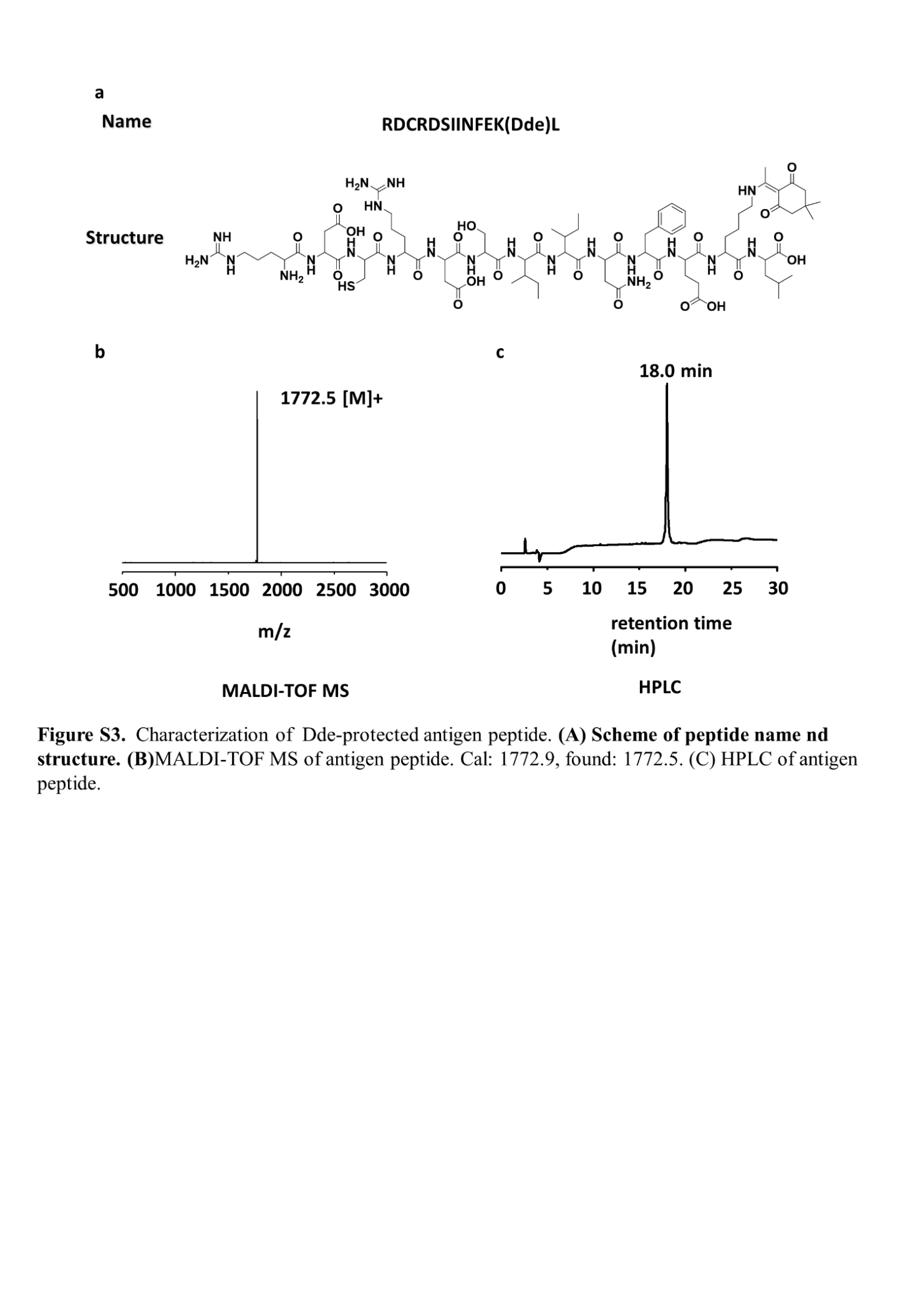


**Figure S2** Peptide antigen structure and characterization. (a) Antigen peptide name and structure. (b) MALDI-TOF MS of antigen peptide. Calcd for C_78_H_125_N_21_O_24_S [M]^+^ 1772.9, found: 1772.5. (c) HPLC of antigen peptide. Column: InertSustain C18 (4.6 × 250 mm, 5 μm). Mobile phase: Acetonitrile-Water (5 : 95 to 75 : 25, v/v, 0 - 30 min); Rate: 1.0 mL/min; Wavelength: 214 nm; Temperature: 25°C.


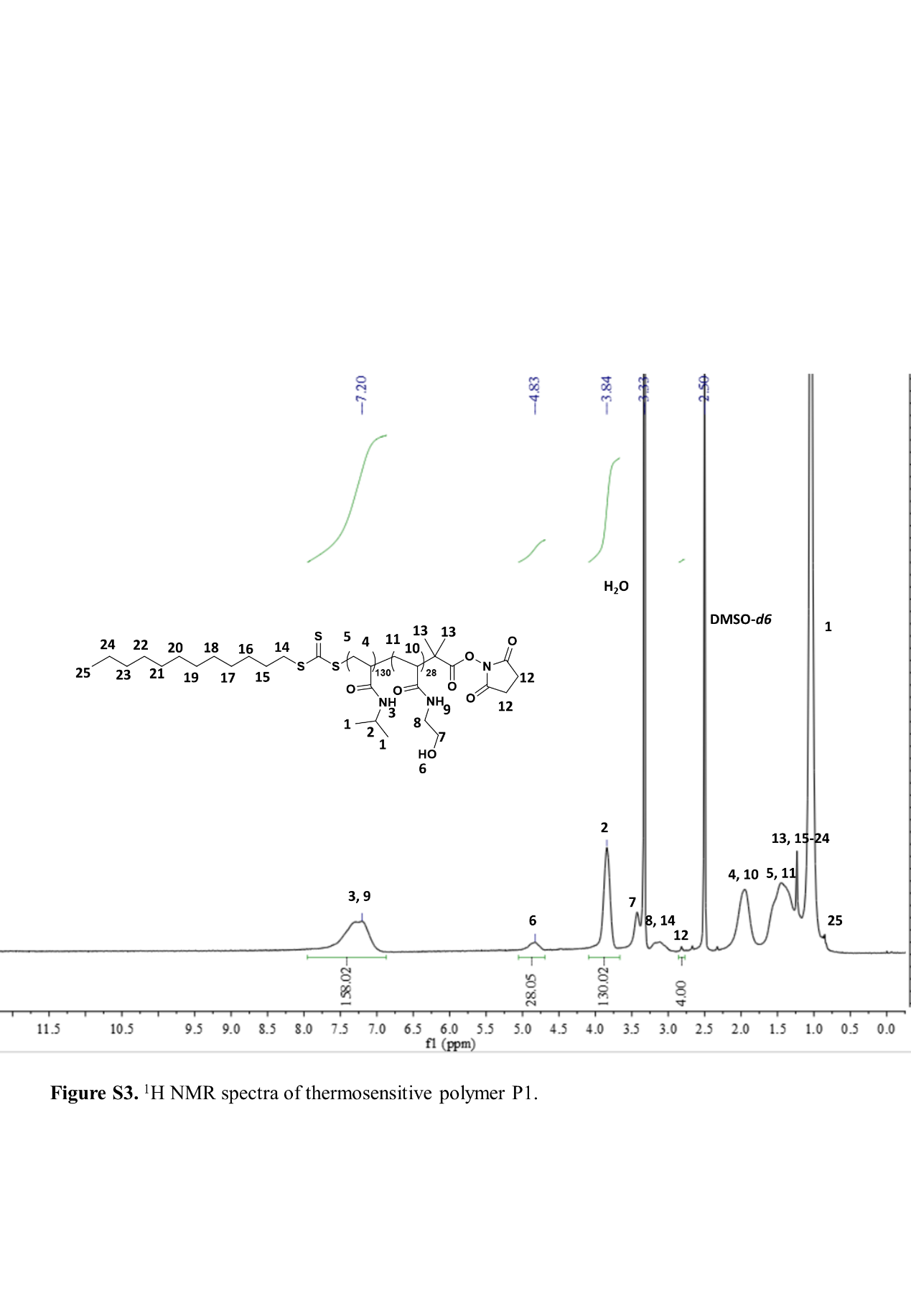


**Figure S3** ^1^H NMR spectra of P1 in DMSO-*d_6_*_._


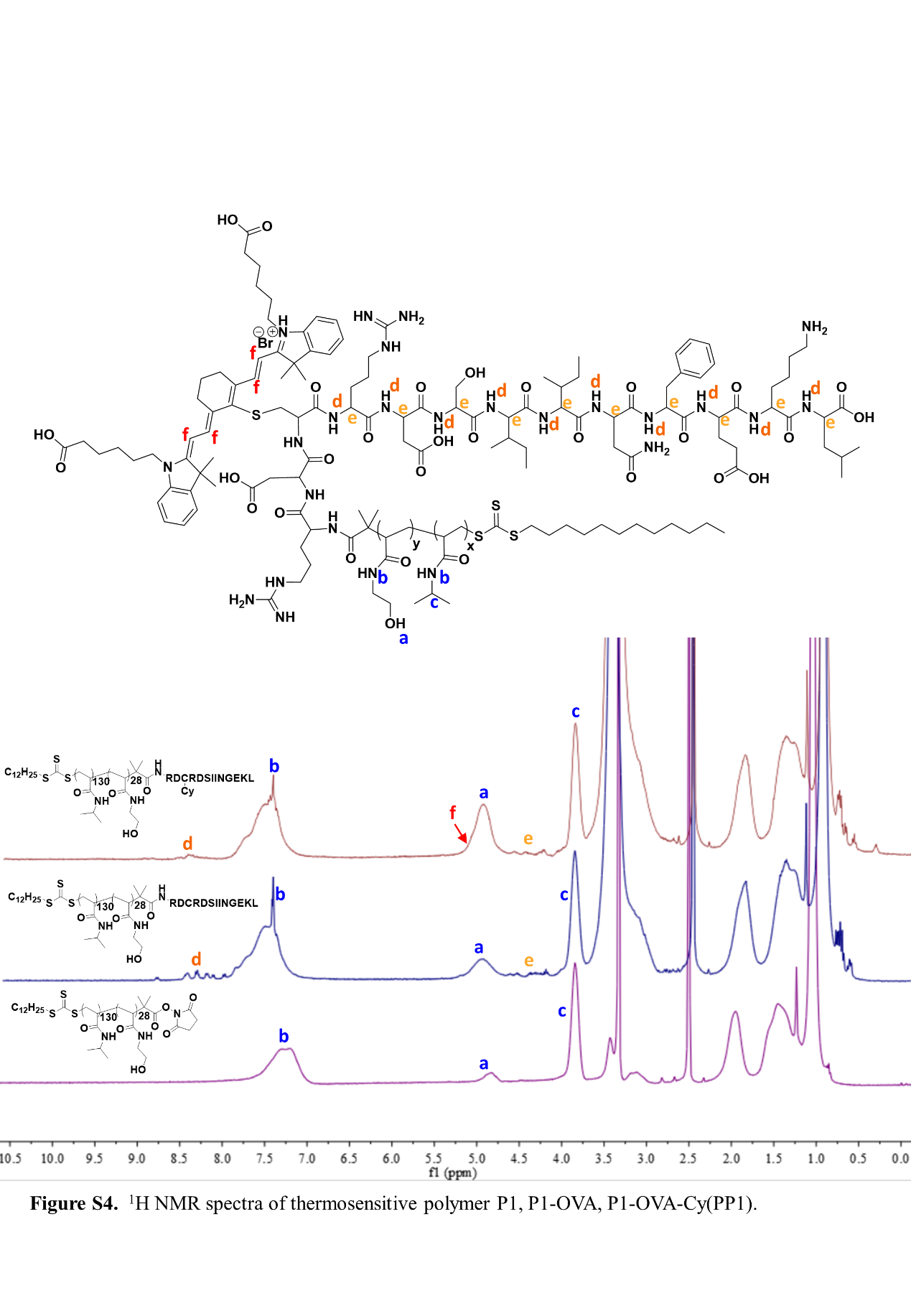


**Figure S4** Structure and ^1^H NMR spectra of P1, P1-OVA, P1-OVA-Cyanine (PP1) in DMSO-*d_6._* Characteristic peaks were labelled as indicated in spectra.


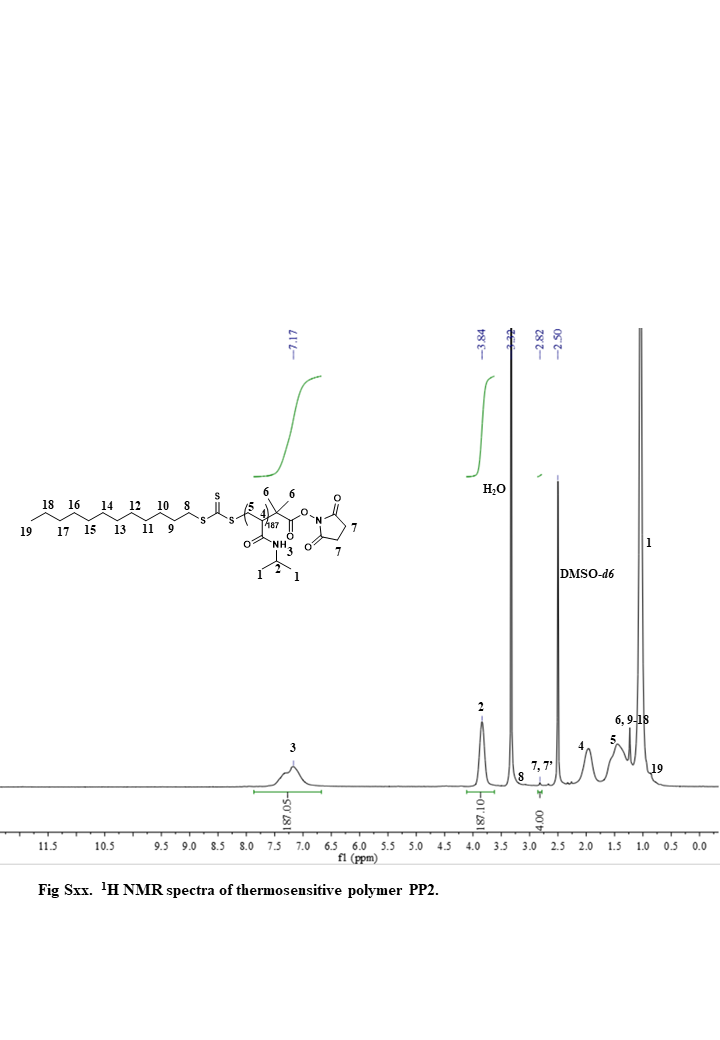


**Figure S5** ^1^H NMR spectra of P2 in DMSO-*d_6._*


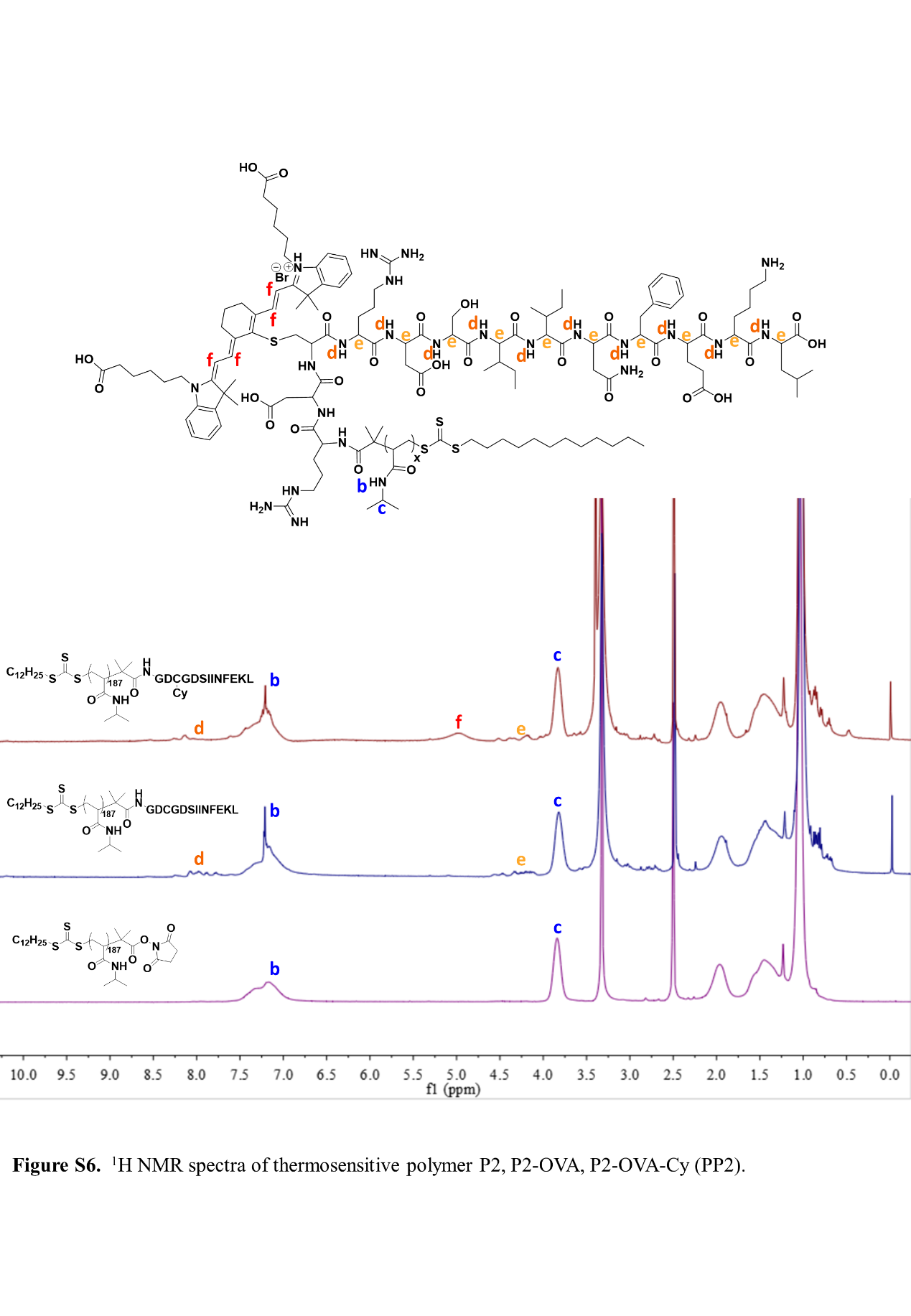


**Figure S6** Structure and ^1^H NMR spectra of P2, P2-OVA, P2-OVA-Cyanine (PP2) in DMSO-*d_6._* Characteristic peaks were labelled as indicated in spectra.


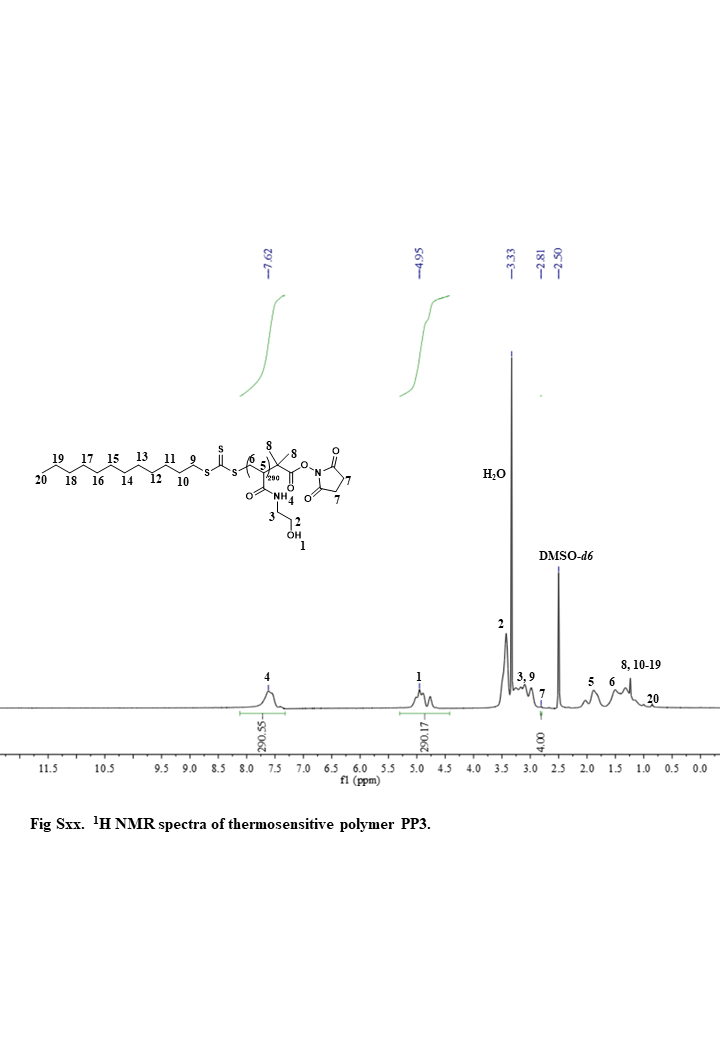


**Figure S7** ^1^H NMR spectra of P3 in DMSO-*d_6._*


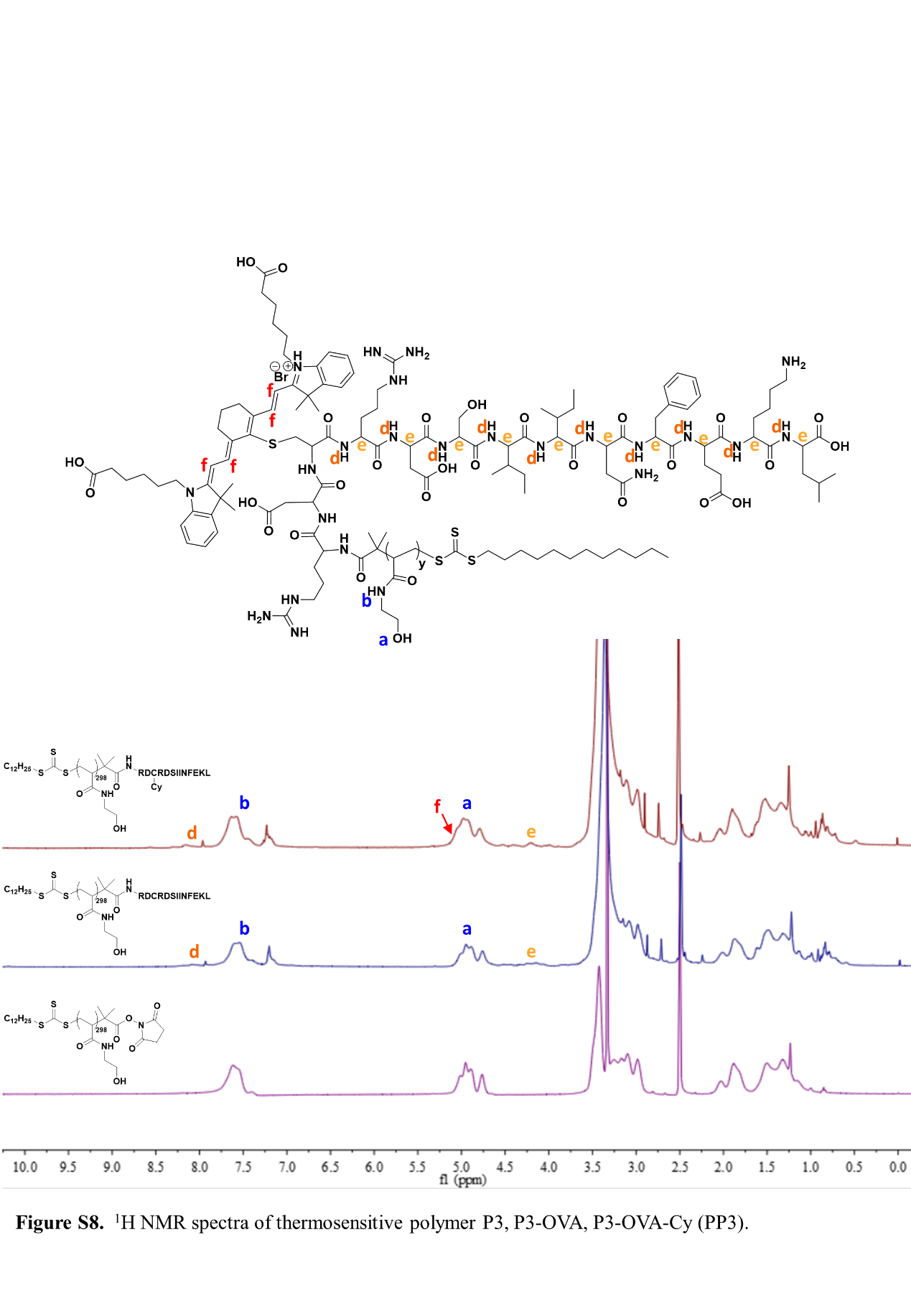


**Figure S8** Structure and ^1^H NMR spectra of P3, P3-OVA, P3-OVA-Cyanine (PP3) in DMSO-*d_6._* Characteristic peaks were labelled as indicated in spectra.


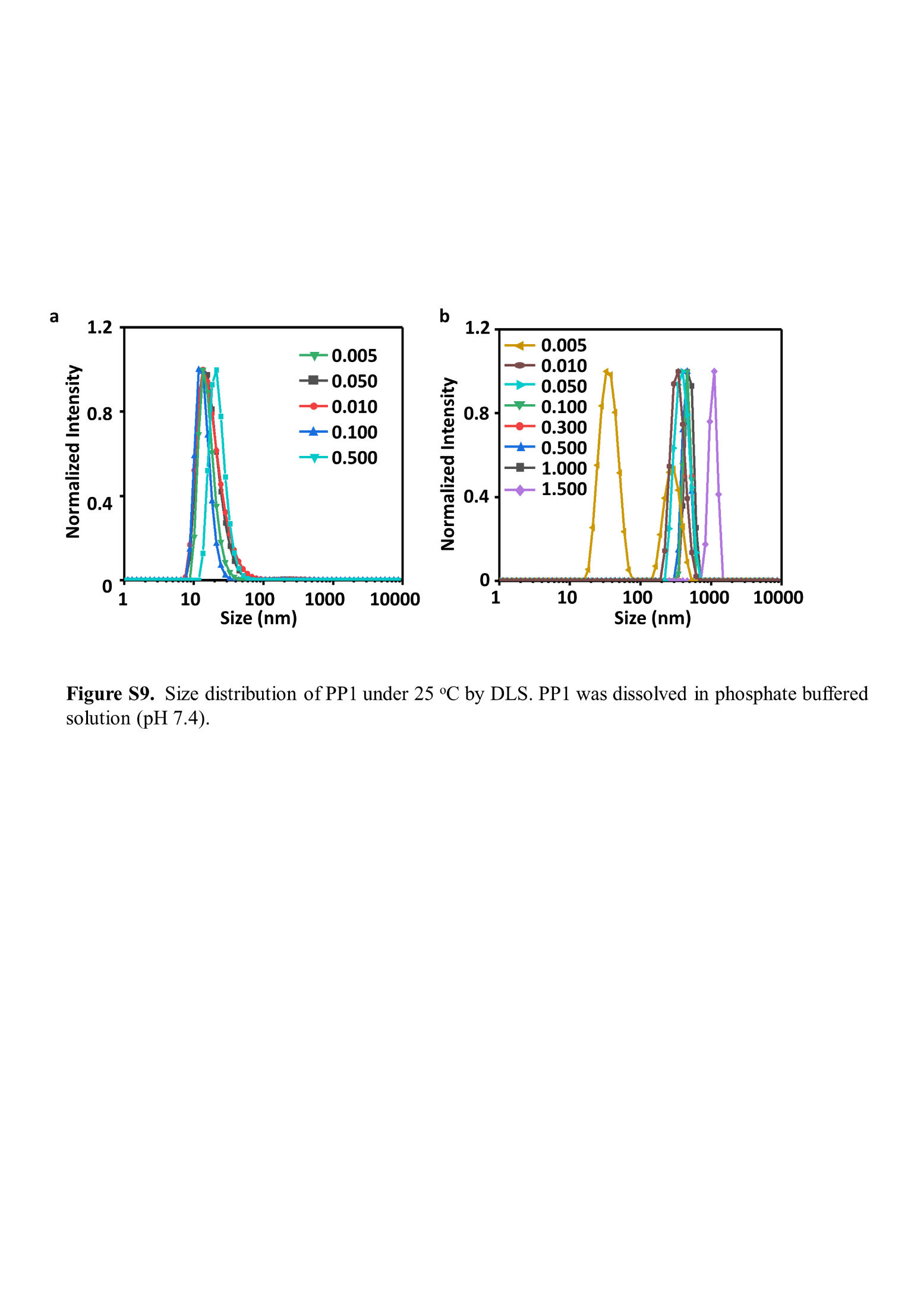

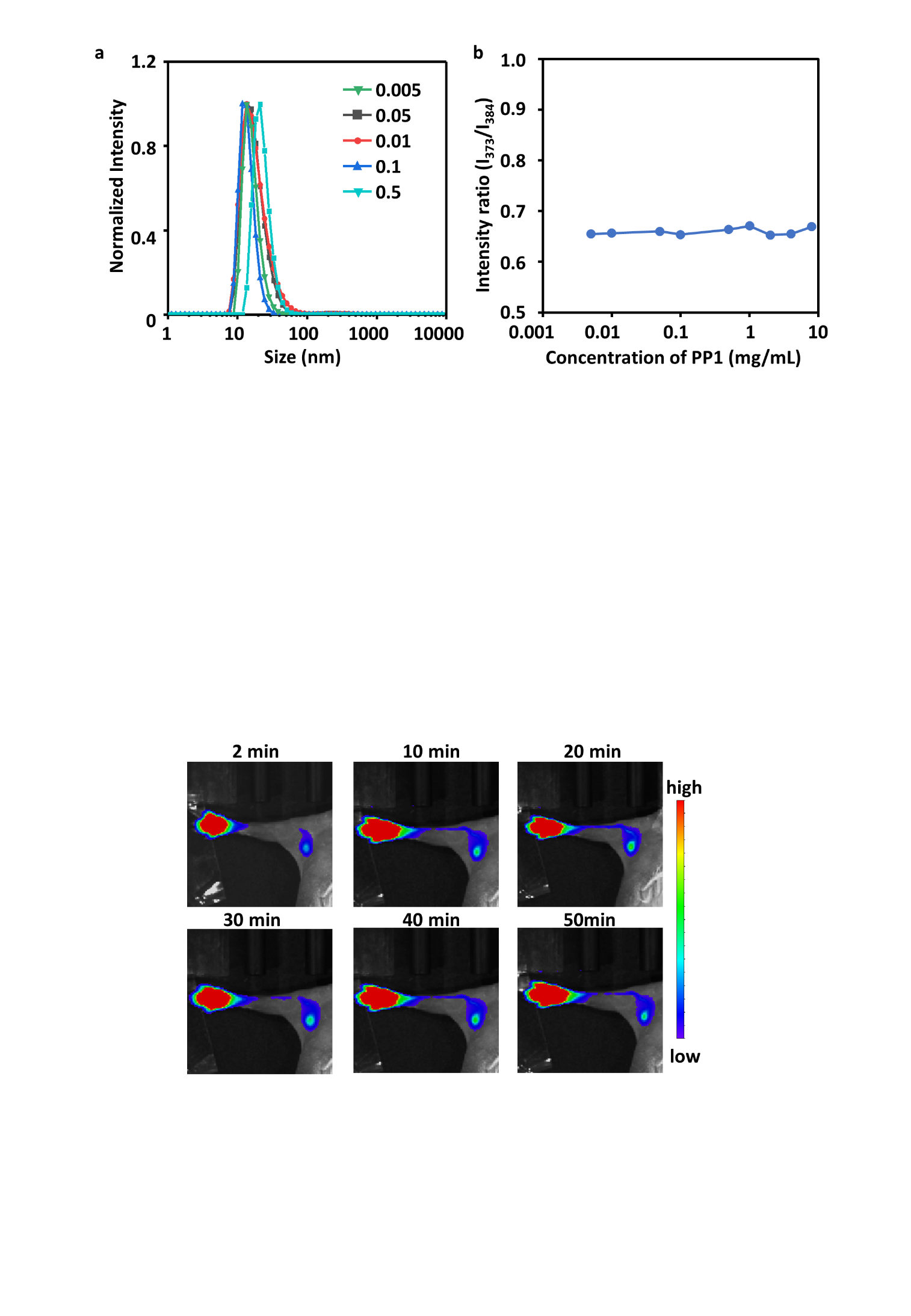


**Figure S9** Size distribution and the critical micelle concentration CMC analysis of PP1 under room temperature. (a) DLS analysis of PP1 with different concentration (mg/mL). (b) CMC analysis of PP1, data were obtained with pyrene as the probe.


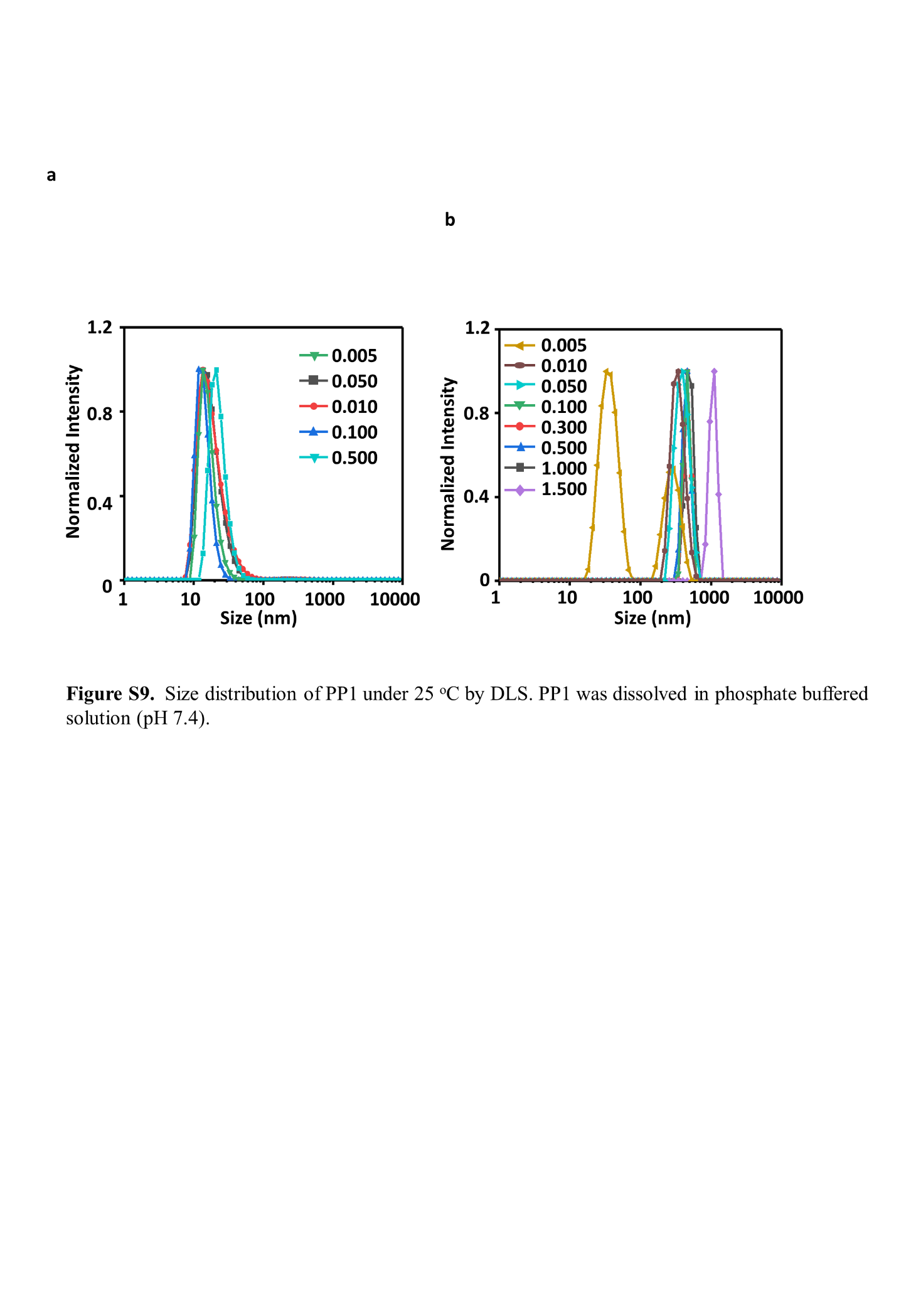


**Figure S10** Size distribution of PP1 under different concentration by DLS. Dispersed solution: PBS; temperature: 41^o^C; The size distribution was nearly unchanged in the range of 0.01-1.0 mg/mL.


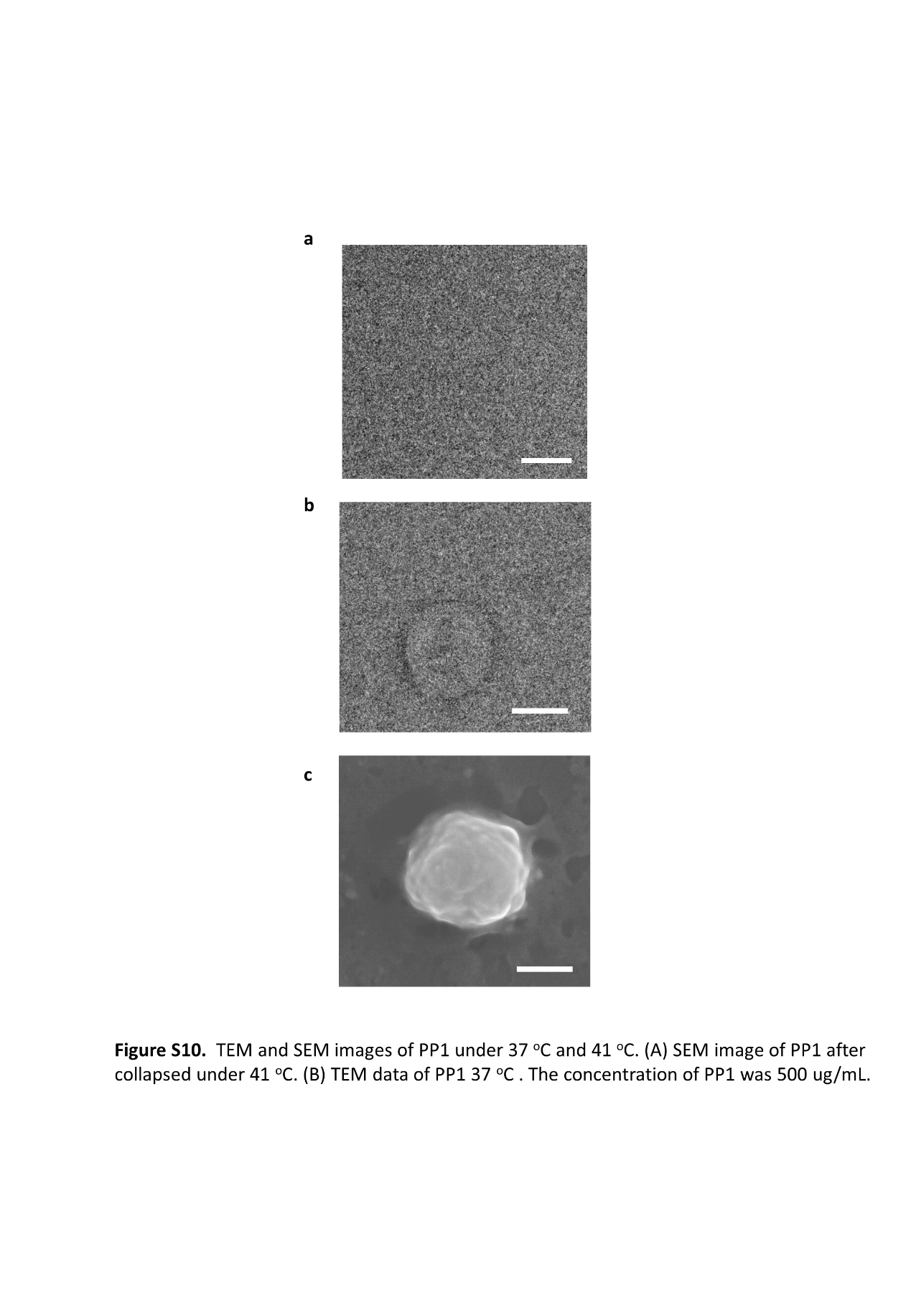


**Figure S11** Morphology characterization of PP1. (a) TEM images of PP1 without phase transition and (b) TEM images of PP1 after phase transition. Scale bar: 0.5 μm. (c) SEM image of PP1 after phase transition. The concentration of PP1 was used at 500 ug/mL. Scale bar: 0.5 μm.


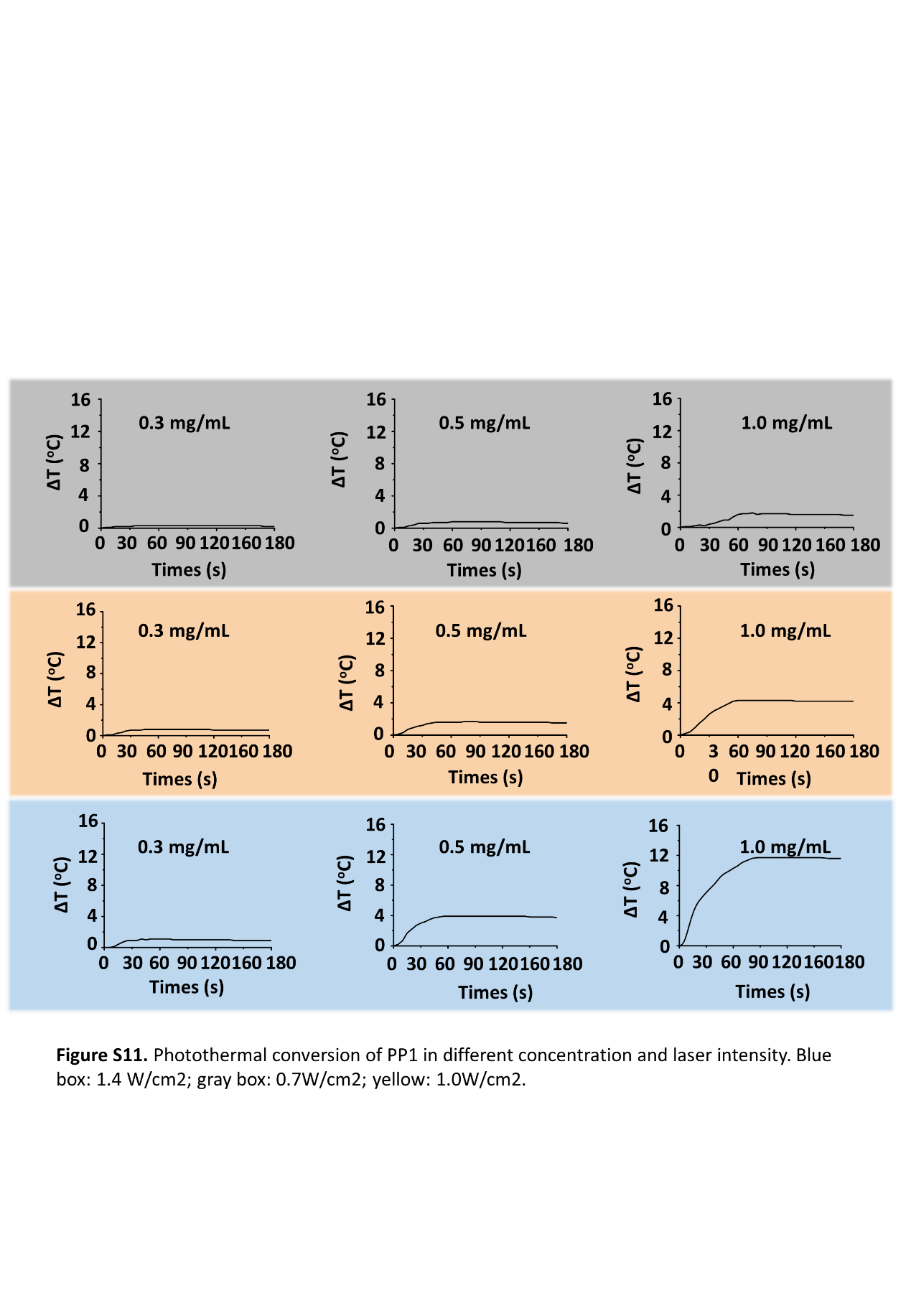


**Figure S12** Photo-thermal conversion under different laser intensity (808 nm) and concentrations of PP1. PP1 was dissolve in PBS and the laser was adjusted with indicated intensity. Gray box: 0.7 W/cm^2^; Yellow: 1.0 W/cm^2^; Blue box: 1.4 W/cm^2^.


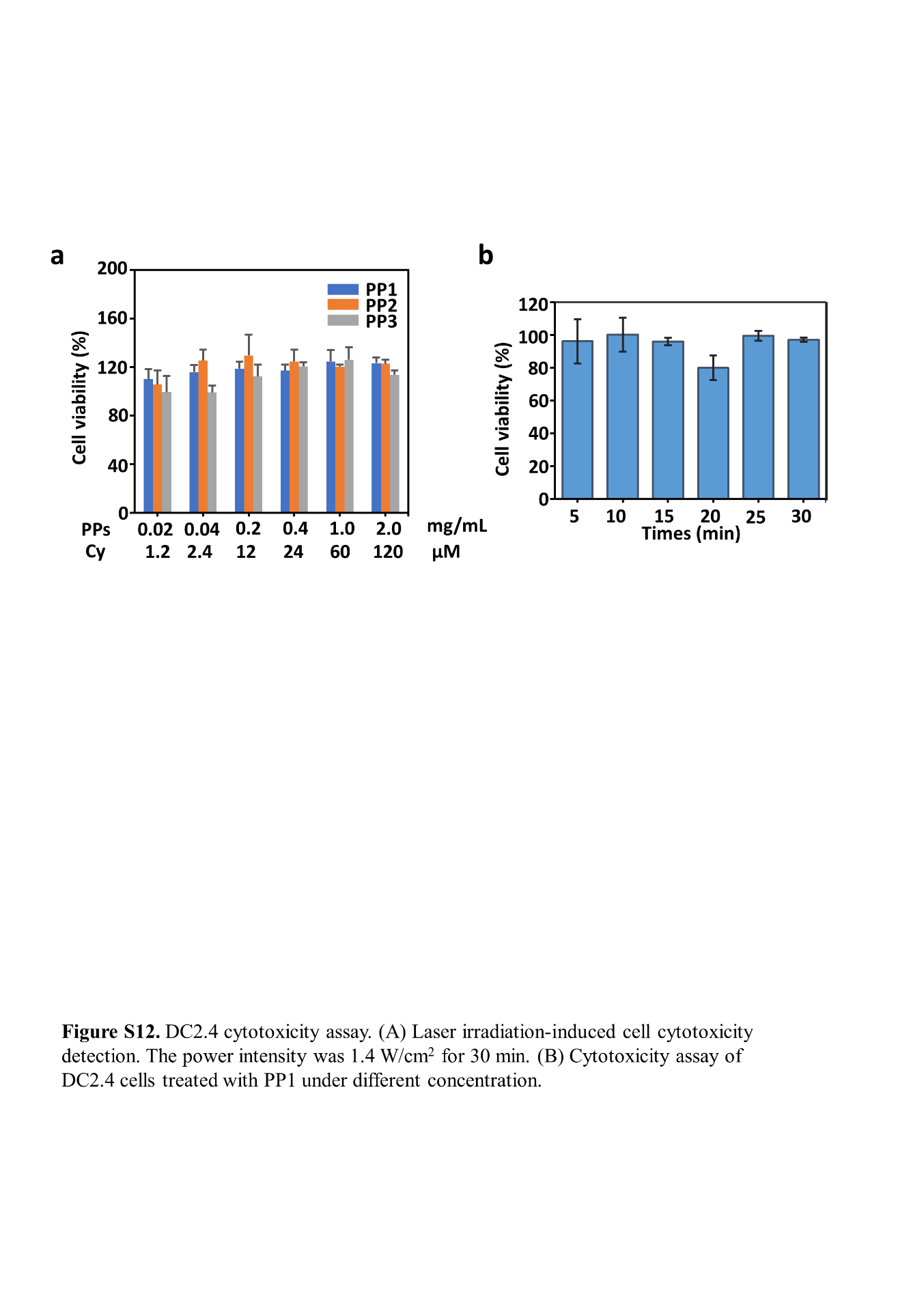


**Figure S13** (a) DC2.4 cell viability under different concentration of PPs and (b) at indicated laser irradiation time (1.4 W/cm^2^, n = 3) for 30 min.


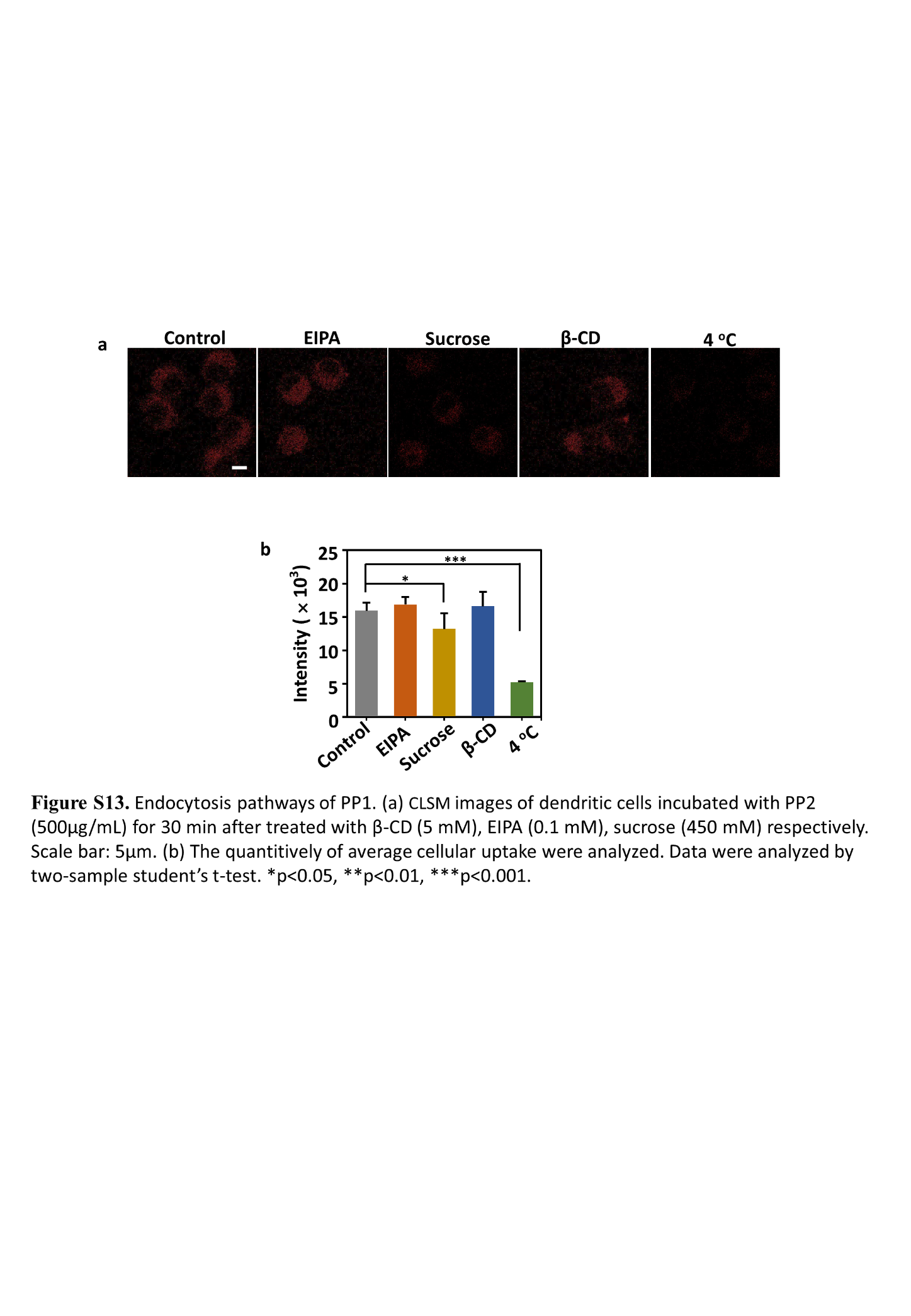


**Figure S14** Endocytosis mechanisms analyzation of transitional cancer vaccines. (a) PP2 uptake (500 μg/mL) detected by CLSM under treatment with different inhibitors. (b) Quantitative data analysis of CLSM images. Data were analyzed by student’s t-test. *p<0.05, ***p<0.001. Scale bar, 5μm.


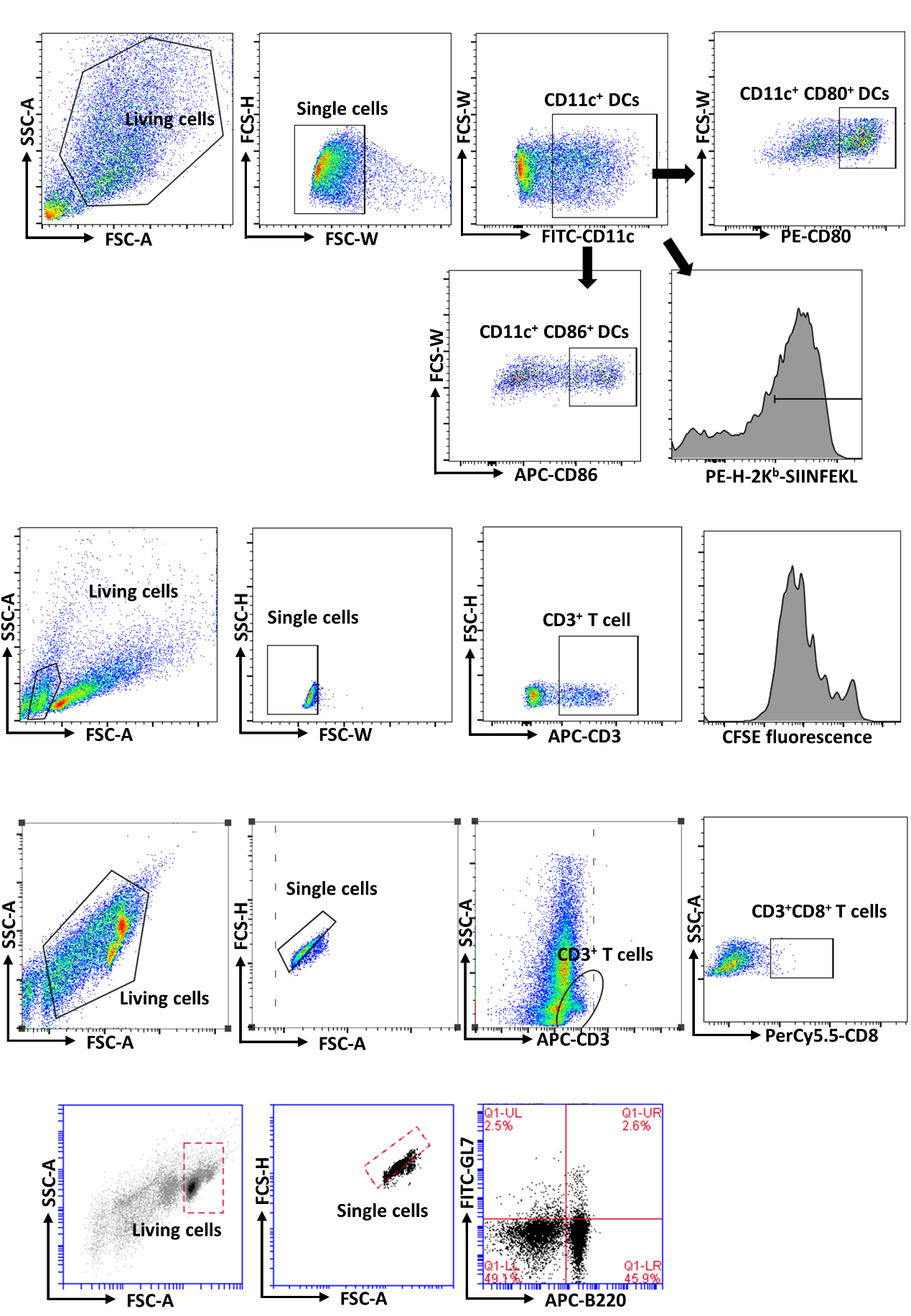


**Figure 15** Sorting strategy of CD80, CD86 expression and OVA-MHC I complex presentation on BMDCs.


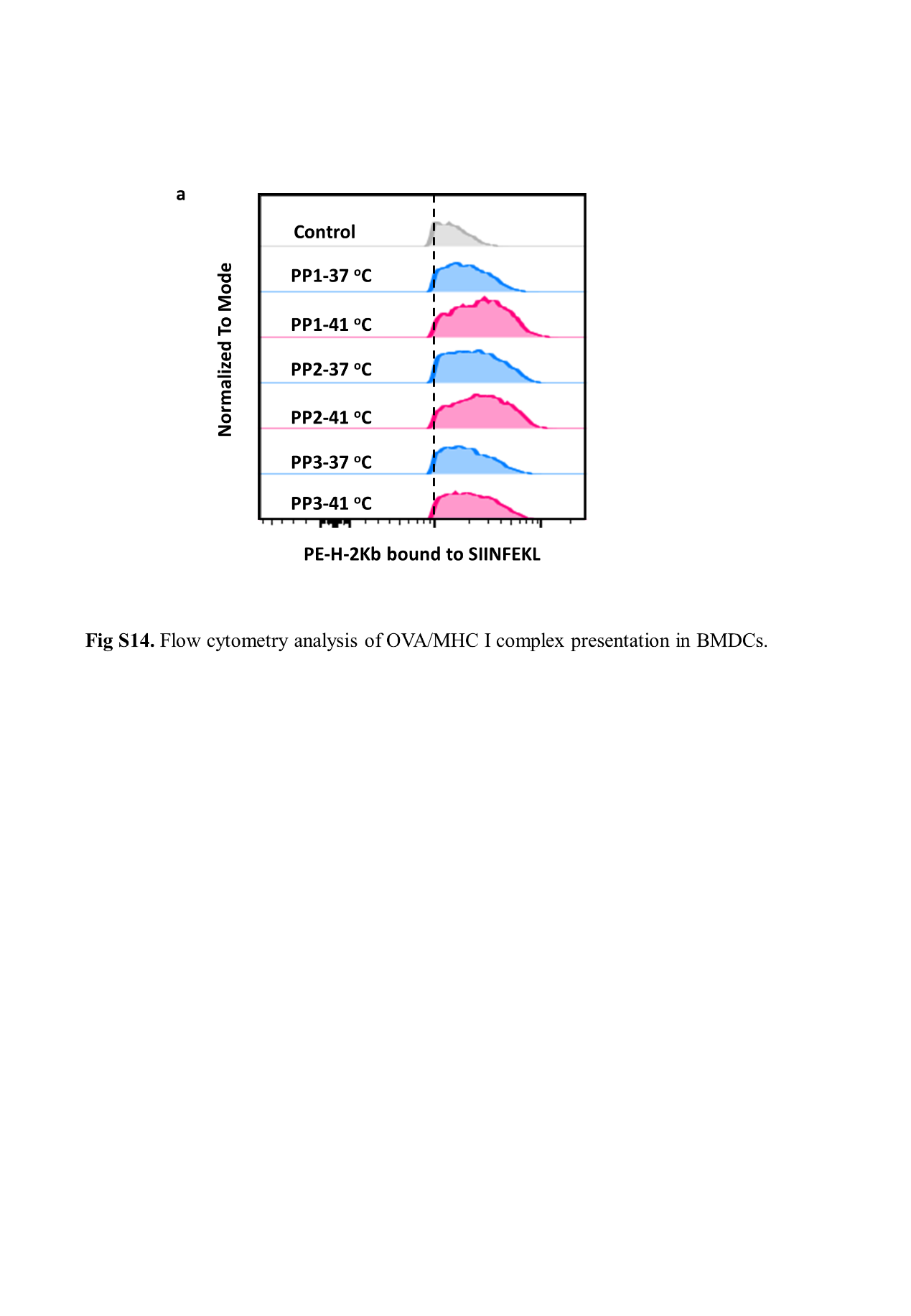


**Figure S16** Flow cytometry analysis of MHC I-OVA antigen complex presentation in BMDCs surface after treated with different PPs. PPs were treated for 30 min, then washed and incubated within RPMI 1640 cell culture medium overnight before going flow cytometry analysis. The right side of dotted line stands for the group of CD11c^+^H-2K^b^-SIINFEKL^+^ BMDCs.


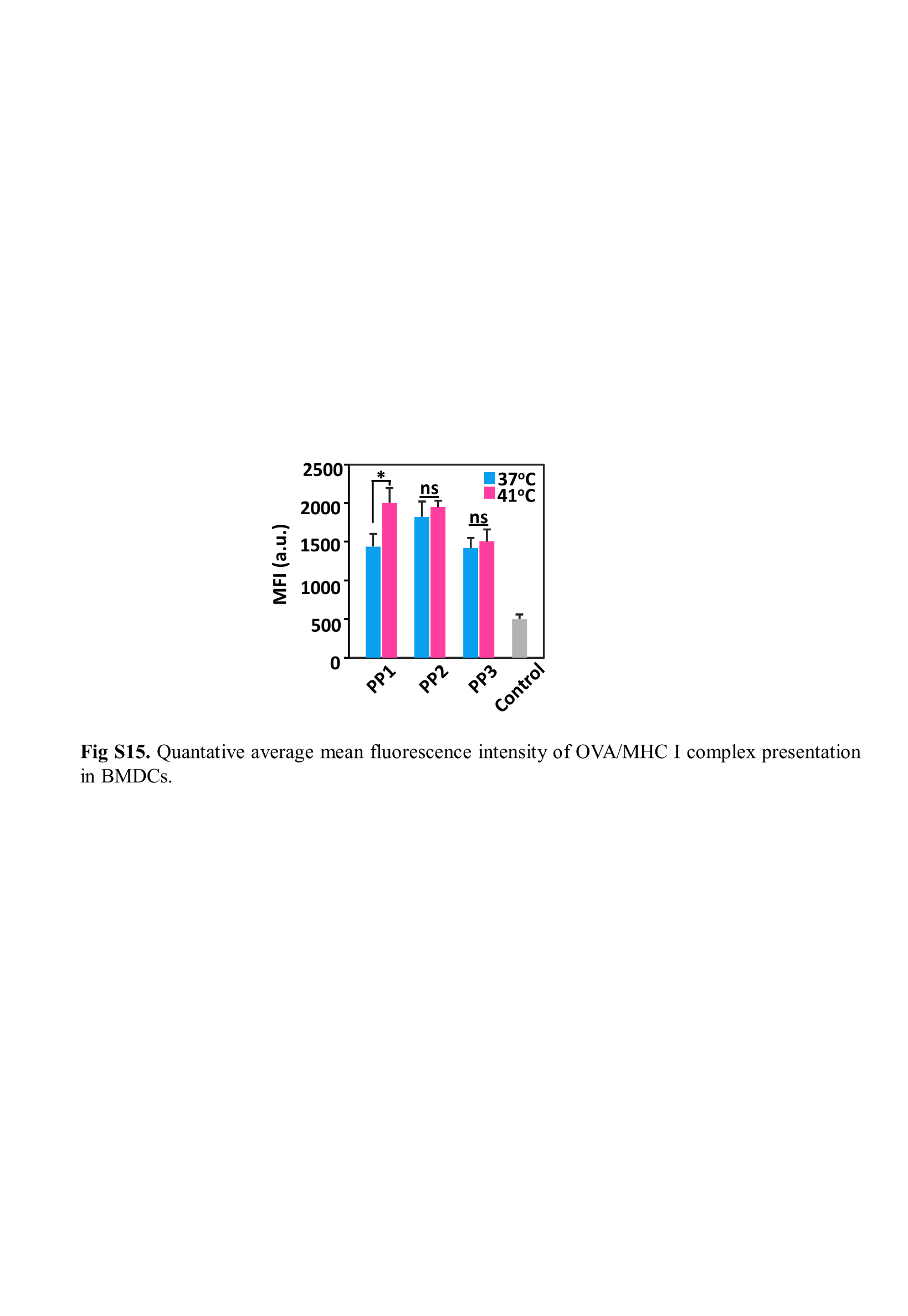


**Figure S17** Quantification analysis by flow cytometry of H-2K^b^-SIINFEKL complex presentation in BMDCs. Data were analyzed based on Fig. S14. the control group was treated with PBS for 30 min, then incubated within RPMI 1640 cell culture medium overnight before going flow cytometry analysis. Data were performed as mean ±S.D. (n=3) and were analyzed by student’s t-test. *p<0.05; ns, not significant.


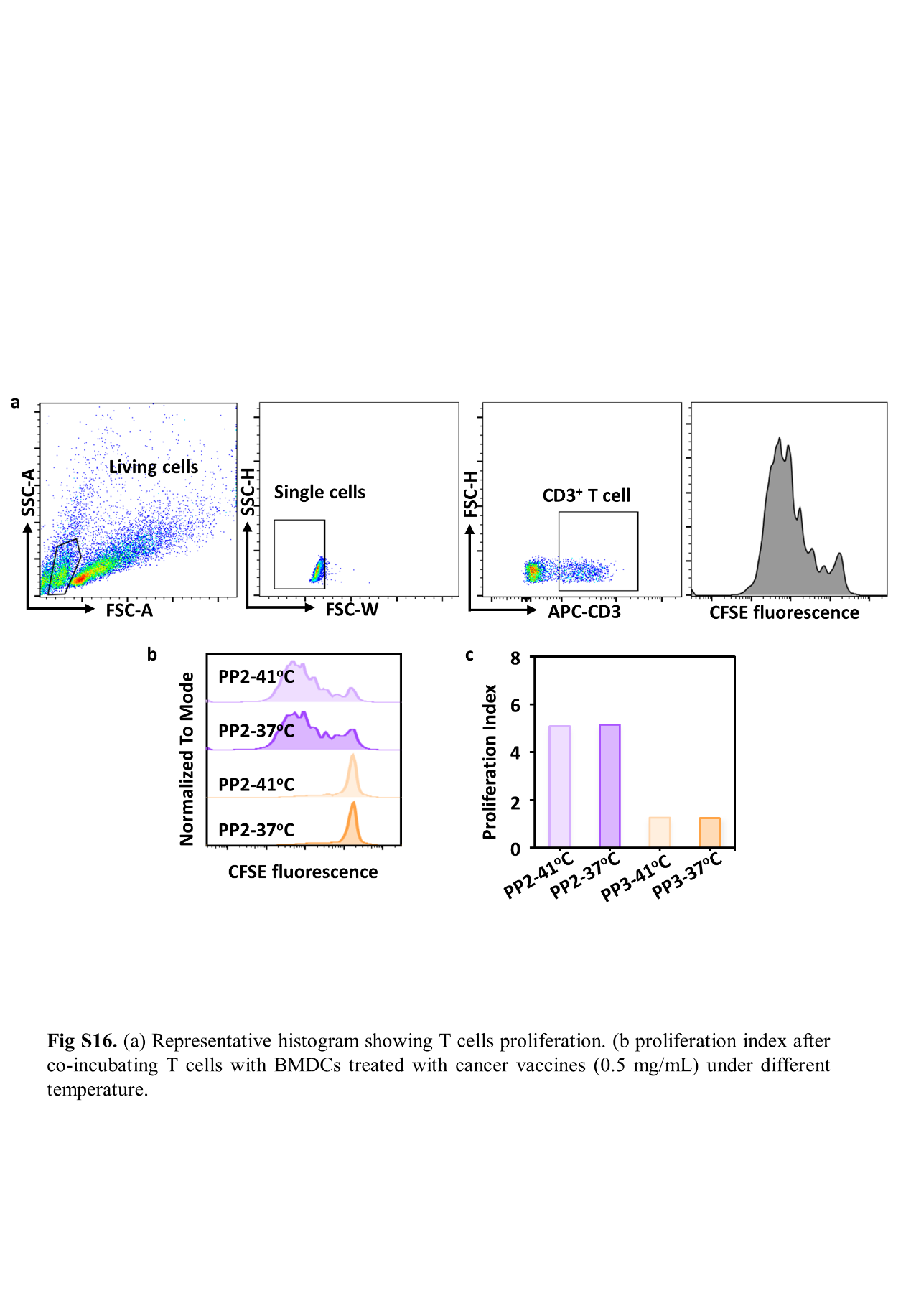


**Figure S18** Representative histogram showing T cells proliferation and proliferation index after co-incubating T cells with BMDCs treated with different PPs (0.5 mg/mL). (a) Sorting strategy of T cell proliferation. (b) Representative histogram of T cells proliferation. (c) Proliferation index of PP1 and PP3 under different temperature.


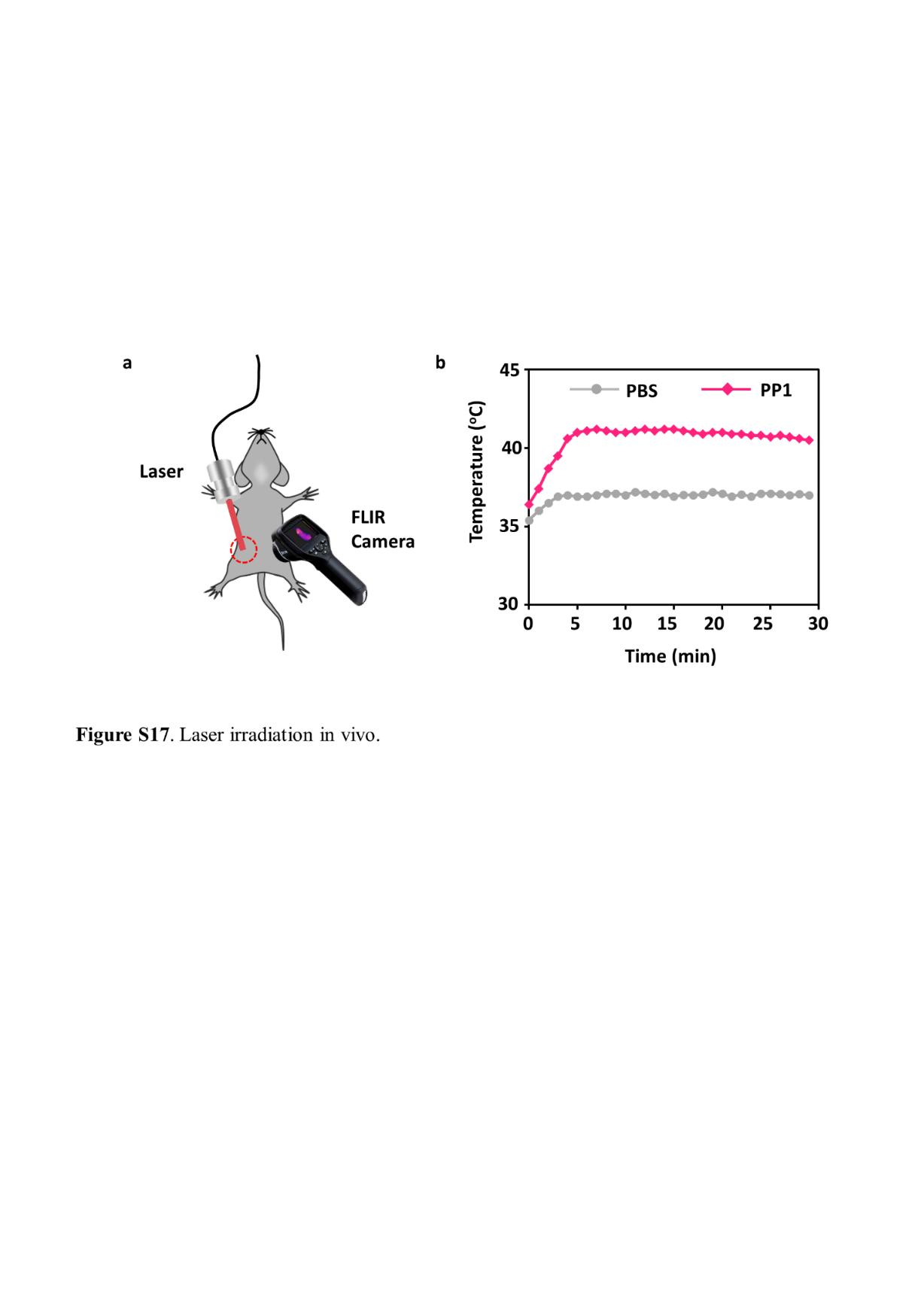


**Figure S19** *In vivo* photo-thermal conversion after injected with PP1 in footpad. The laser (1.4 W /cm^2^) was irradiated at inguinal lymph node for 10 min and monitored with FLIR camera. (a) Schematic illustration of laser-induced temperature enhancement. (b) Temperature changes monitoring by FLIR camera.

**
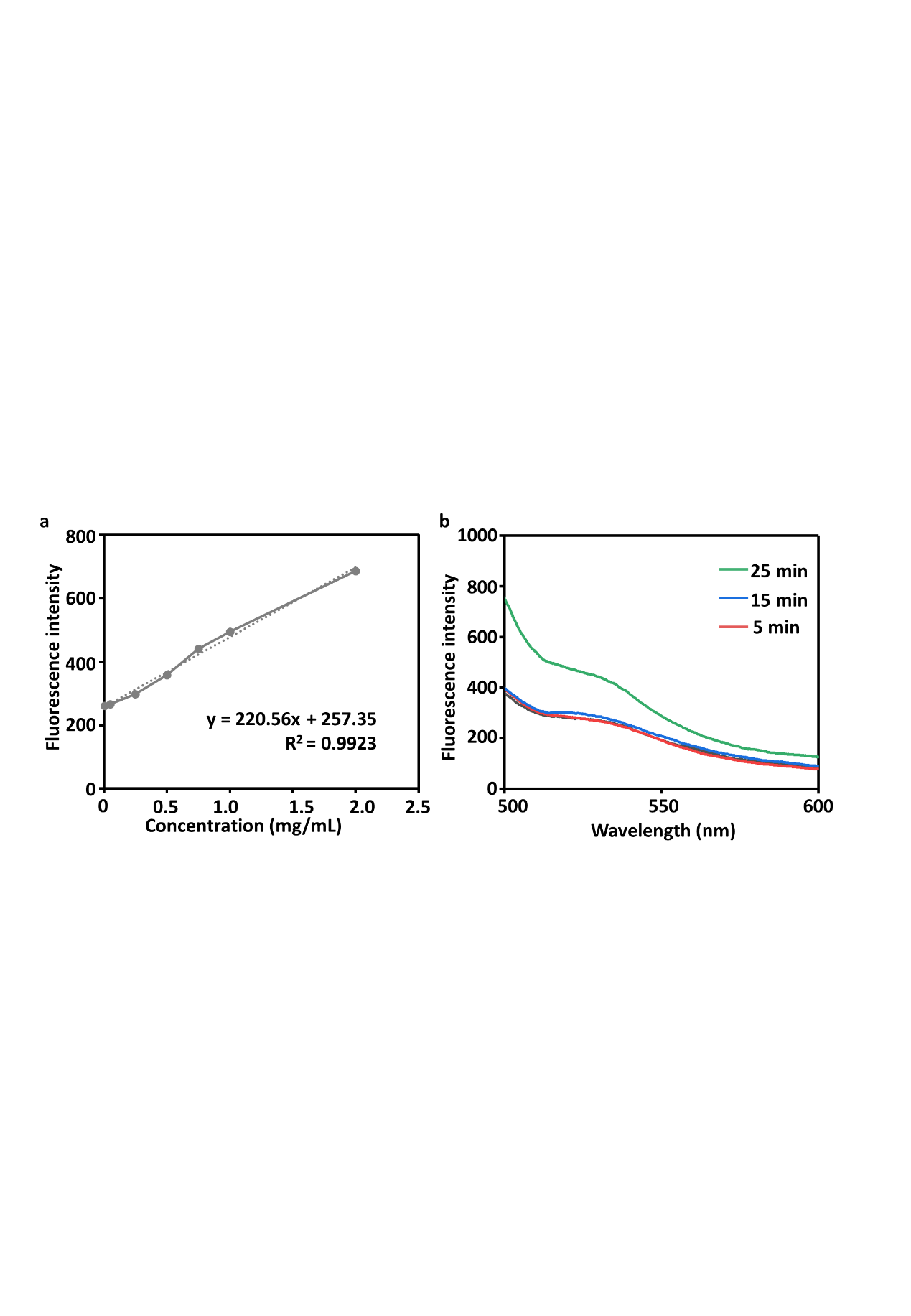
**

**Figure S20**. (a) NBD fluorescence was obtained at 25 min. (b) NBD fluorescence in lymph node supernatant in different time. Mice were injected 4-Chloro-7-nitro-1,2,3-benzoxadiazole (NBD) conjugated PP1(5 mg/ml) at footpad. The inguinal lymph node was dissected and homogenized in 0.1 mL PBS. Supernatant was collected and put laser irradiation for 1 min and detect the NBD fluorescence at λ_Ex_=460 nm.


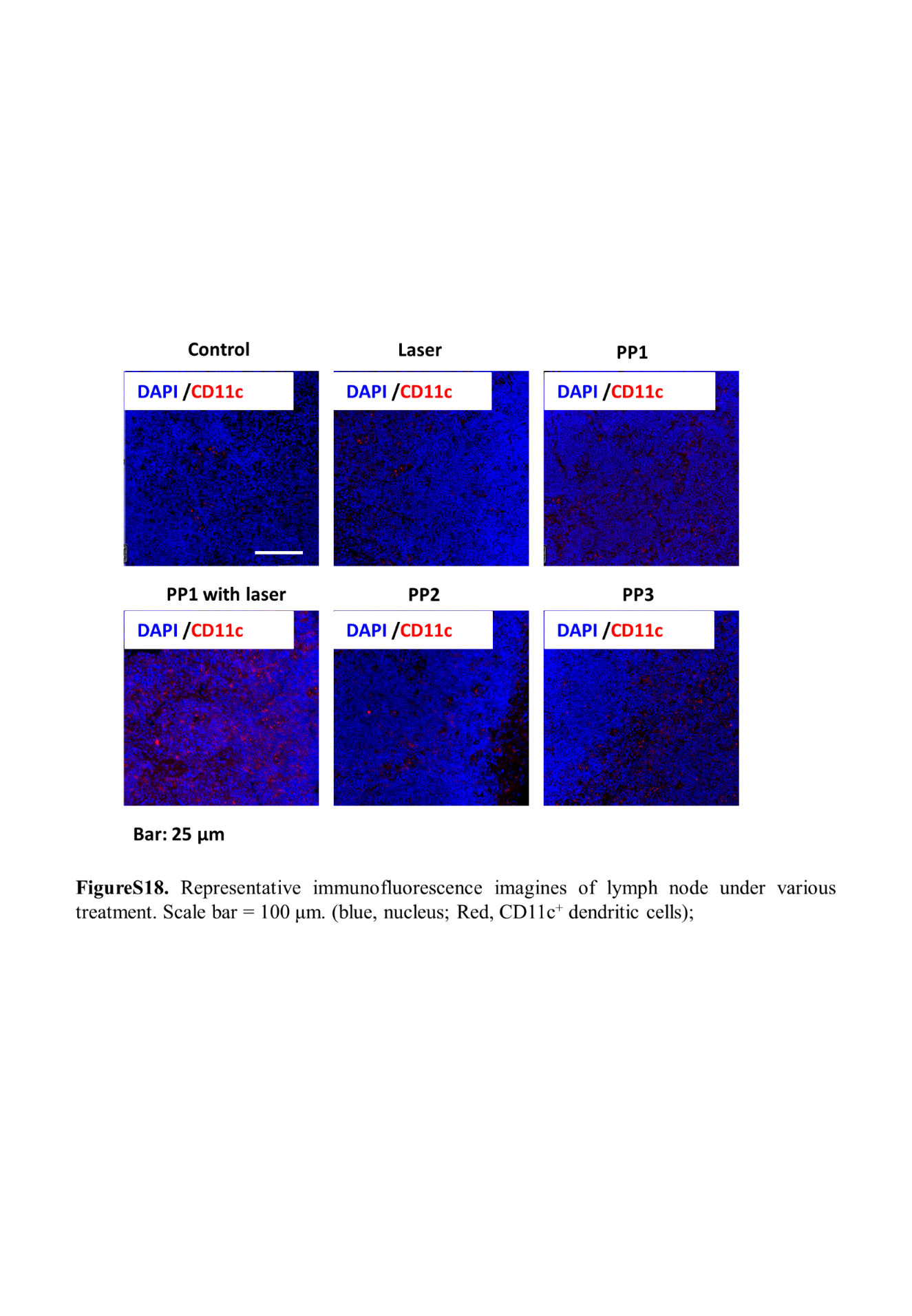


**Figure S21** Representative immunofluorescence images of lymph node under various treatment. (blue, nucleus; Red, CD11c^+^); Bar: 25μm.


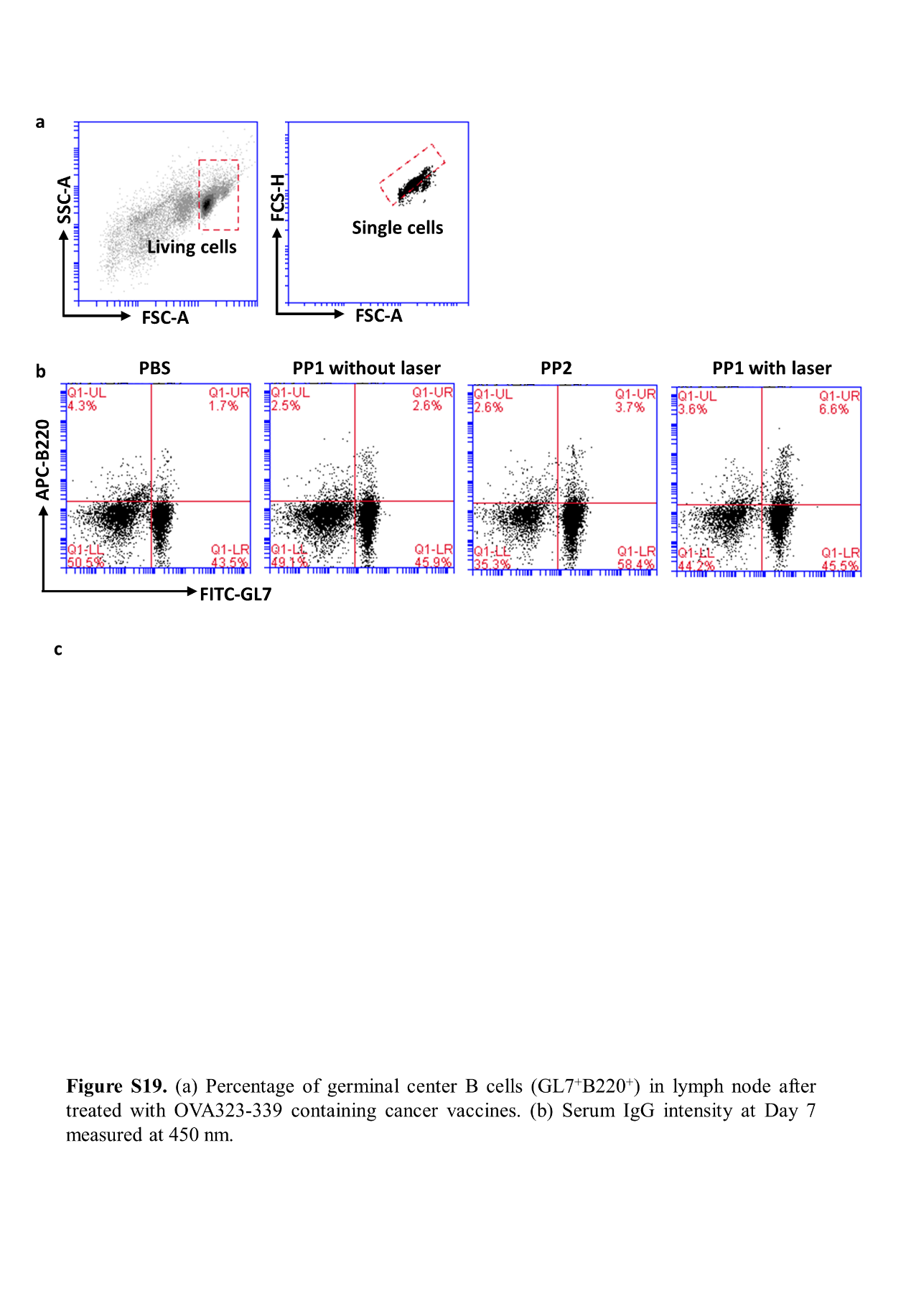


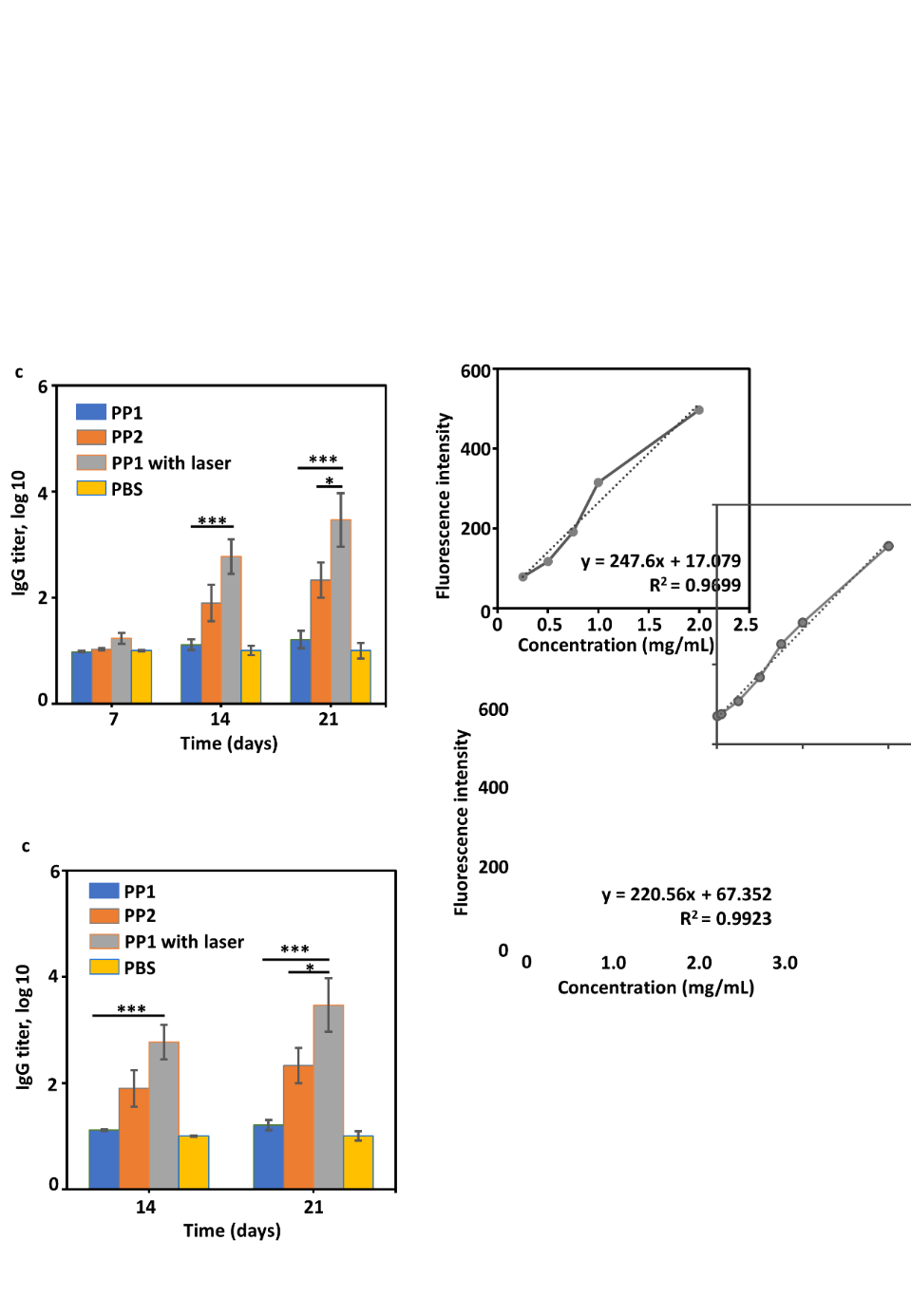


**Figure S22** Humoral immunity induced by cancer vaccines. (a) Sorting strategy of germinal center B cells (GL7^+^B220^+^). (b) Percentage of germinal center B cells (GL7^+^B220^+^) in lymph node after treated with OVA_323-339_ linked cancer vaccines. (c) Serum IgG intensity at day 7, day 14 and day 21 measured at 450 nm. Data were performed as mean ± S.D. (n=3) and were analyzed by student’s t-test. *p<0.05; ***p<0.001.


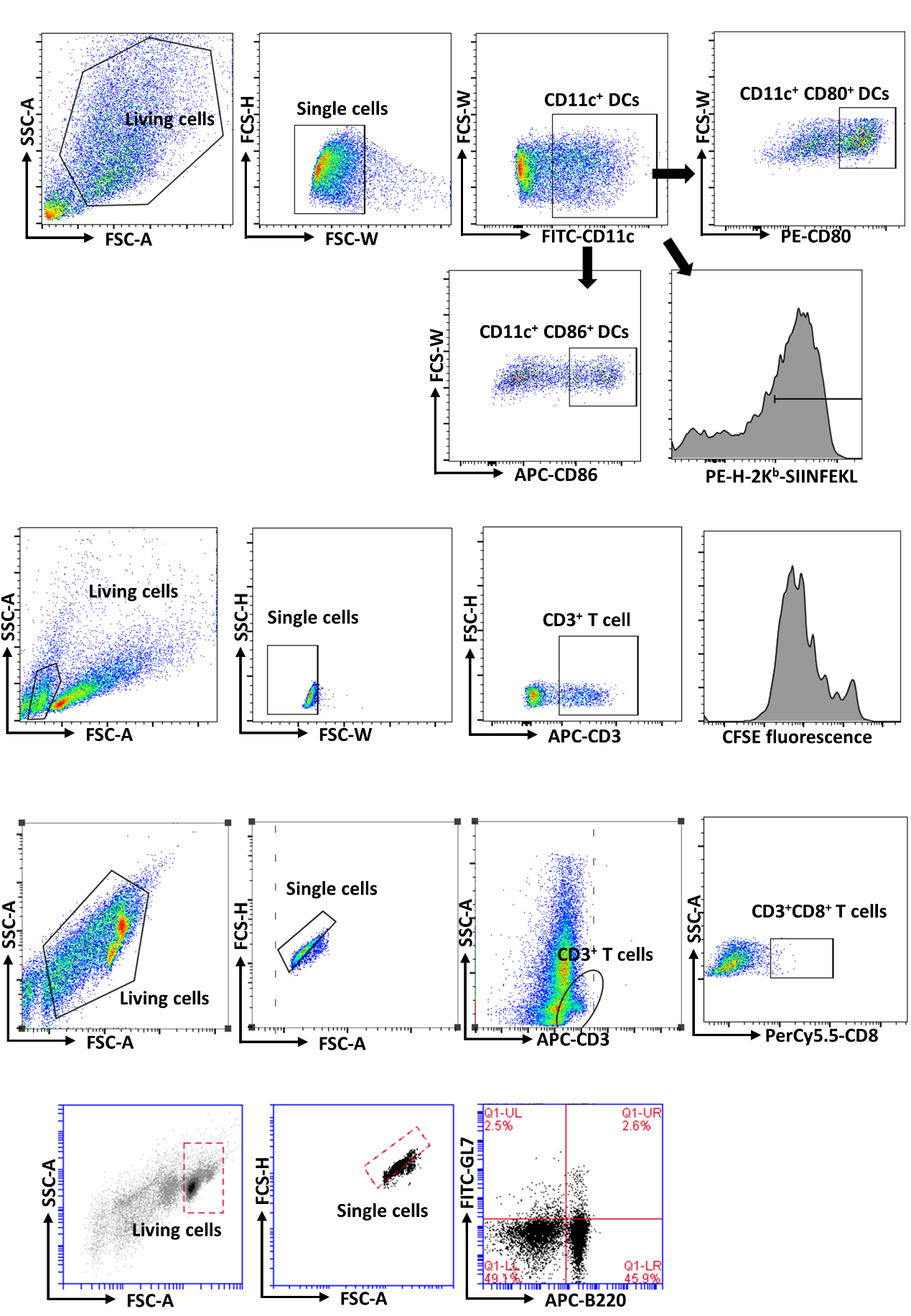


**Figure S23** Sorting strategy of CD3^+^CD8^+^ T cells in tumor tissue.


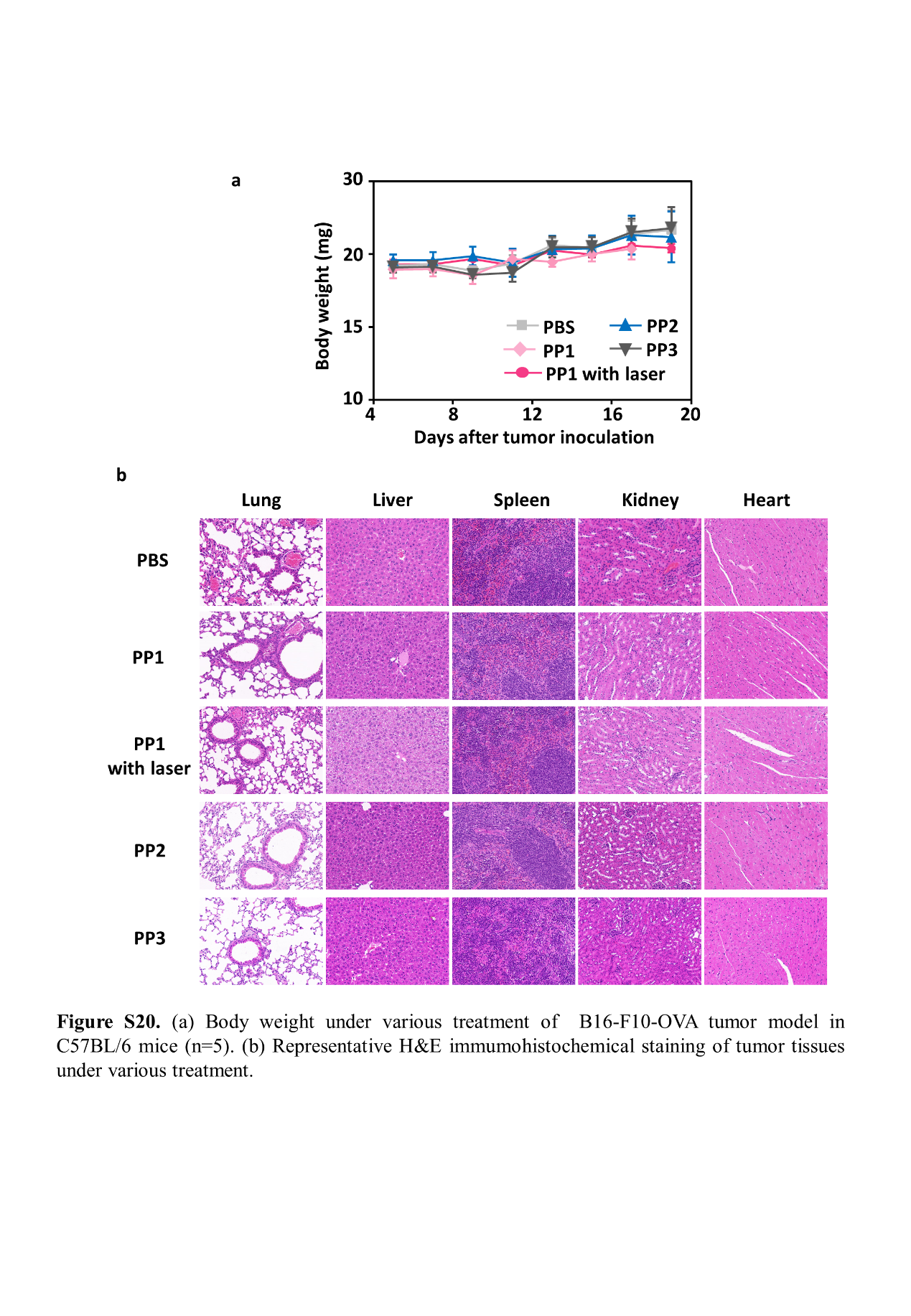


**Figure S23** Body weight and major organs toxicity analysis. (a) Body weight of mice treated with PBS, PP1, PP2, PP3 and PP1 with laser (1.4 W/cm^2^ for 30 min). (b) The histopathology and hematology (H&E) of PBS, PP1 with laser (1.4 W/cm^2^ for 30 min), PP1, PP2, PP3 treatment group staining of heart, liver, spleen, kidney, and lung. (magnification: ×20)
